# Metal–organic tube or layered assembly: reversible sheet-to-tube transformation and adaptive recognition†

**DOI:** 10.1039/d0sc01176b

**Published:** 2020-08-12

**Authors:** Jiayue Tian, Luyao Liu, Kang Zhou, Zixiao Hong, Qihui Chen, Feilong Jiang, Daqiang Yuan, Qingfu Sun, Maochun Hong

**Affiliations:** State Key Laboratory of Structure Chemistry, Fujian Institute of Research on the Structure of Matter, Chinese Academy of Sciences Fuzhou Fujian 350002 China chenqh@fjirsm.ac.cn hmc@fjirsm.ac.cn; Institute of Urban Environment, Chinese Academy of Sciences Xiamen 361021 China; University of the Chinese Academy of Sciences Beijing 100049 China; Zhengzhou University of Light Industry Zhengzhou 450001 P. R. China

## Abstract

Rational preparation of an adaptive cavity-like enzyme is a great challenge for chemists. Herein, a new self-assembly strategy for the rational preparation of metal–organic tubes with nano-channels has been developed; both 1D metal–organic tube and corresponding 2D layered assemblies can be selectively synthesized driven by different templates; reversible sheet-to-tube transformation can be realized and the key intermediate has been identified. Furthermore, the newly formed nano-channel displays excellent polarity-selectivity for encapsulation of guest molecules, and can be further expanded or contracted through guest-driven adaptive deformation; even induced by very similar guest molecules, the adaptive deformations can also be obviously distinguished. Finally, the key chemicals benzene/hexane with a similar size can also be effectively separated by such nano-channels in the liquid phase. Our work not only provides a new synthetic strategy for the rational synthesis of metal–organic tubes with a reversible sheet-to-tube transformation character, but also gives a potential method for the construction of adaptive host-like enzymes and an in-depth understanding of the nature of adaptive host and guest molecules.

## Abstract 2

A new strategy for preparing metal–organic tubes with reversible sheet-to-tube transformation character has been developed. The new-formed tube can selectively recognize and separate guest molecules with similar size.

## Graphical abstract

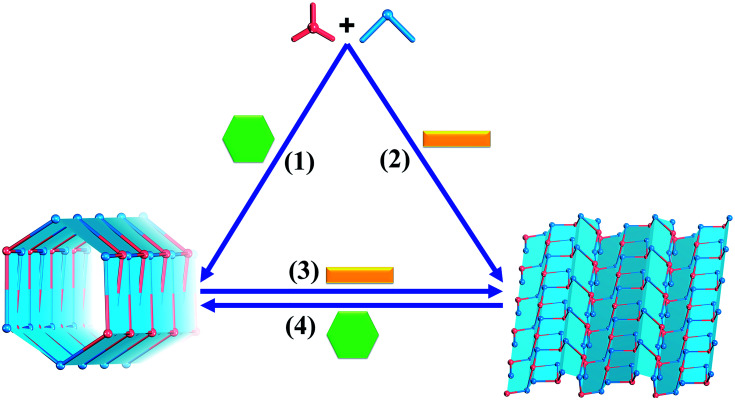

## Introduction

Adaptive deformation of enzymes has been speculated to play a key role in enzyme catalysis, in which the conformational variation of the enzyme will lead to an increase in reaction activity and selectivity.^1^ Inspired by this, rational design and synthesis of adaptive cavities that can respond to external stimuli have attracted more and more attention.^2–14^ However, synthesis of adaptive cavities that can clearly distinguish specific guest molecules with a similar size as enzymes remains a big challenge.

Since the first discovery of carbon nanotubes by Iijima in 1991,^15^ considerable attention has been paid to the efficient synthesis of various tubular materials.^16–23^ As a new class of discrete tubular materials, infinite one-dimensional (1D) metal–organic tubes constructed from metal ions and organic ligands are still in their infancy.^24,25^ To date, only a few metal–organic tubes have been prepared.^22,26–52^ Most of those studies mainly focused on structural characterization; only limited applications such as selective adsorption and catalysis have been developed;^31,37,38^ how to use the confinement effect of nano-channels of metal–organic tubes to discover unique physical^27^ or chemical properties remains a big challenge. Moreover, inspired by the reversible sheet-to-tube transformation between 1D carbon nanotubes and two-dimensional (2D) graphene,^53,54^ chemists seek to realize such reversible transformation in the metal–organic tube area;^44,45,51^ however, the reversible sheet-to-tube transformation based on the same building unit like that of carbon nanotubes and graphene has never been realized. Indeed, such a procedure is highly challenging, which requires firstly deconstructing a large number of chemical bonds contained in a given structure and then orderly reconstructing them into the corresponding isomer. All of these inspire chemists to develop new strategies for the rational construction of metal–organic tubes and to gain further insight into their charming nano-channels.

Coordination driven self-assembly has been proved to be a highly useful approach for the preparation of well-defined supramolecular assemblies due to its good predictability; where metal ions with specific coordination geometries serve as acceptors and organic ligands serve as donors. So far, many elegant molecular coordination tubes^31,55–63^ and a number of dynamic self-assembly systems driven by different external stimuli^64–67^ have been developed and investigated. So using dynamic coordination self-assembly to prepare both tubular and layered metal–organic assemblies should be reasonable.

**Fig. 1:**
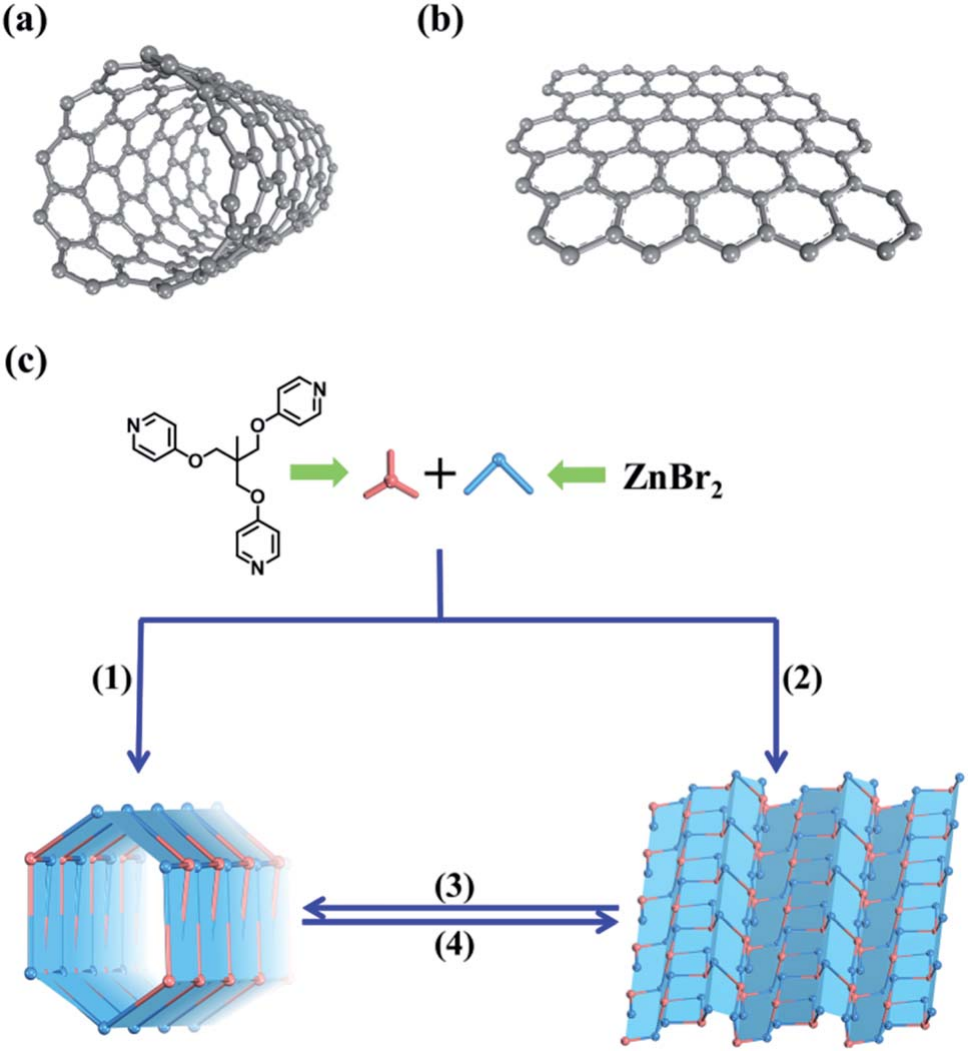
(a) Carbon nanotubes. (b) Graphene. (c) Selective self-assembly and reversible transformation of both the 1D tubular structure and 2D sheet structure from the same organic ligands and metal sources. Pink building block represents the TPOME ligand, and the blue building block represents the zinc bromide unit. (1)–(4) represent DMPU driven self-assembly of the 1D tubular structure, CH_3_CN driven self-assembly of the 2D sheet structure, DMPU driven transformation of the 2D sheet structure into the 1D tubular structure, and CH_3_CN driven transformation of the 1D tubular structure into the 2D sheet structure, respectively.

**Fig. 2:**
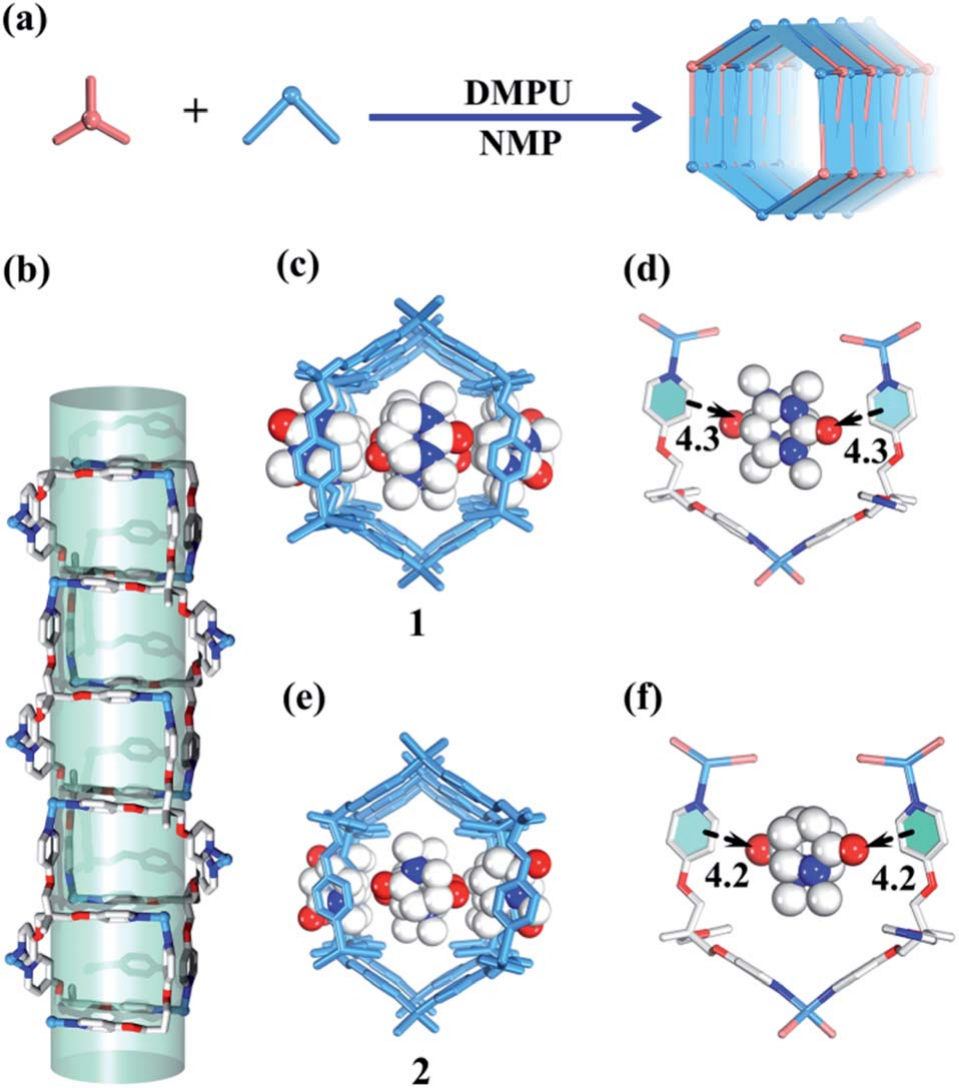
(a) Guest-driven assembly of 1D metal–organic tubes with nano-channels. (b) The tubular structure is comprised of **1** and **2**. (c) The structure of **1**. (d) The template effect of DMPU molecules. (e) The structure of **2**. (f) The template effect of NMP molecules (blue/white/red balls represent N/C/O atoms respectively).

**Fig. 3:**
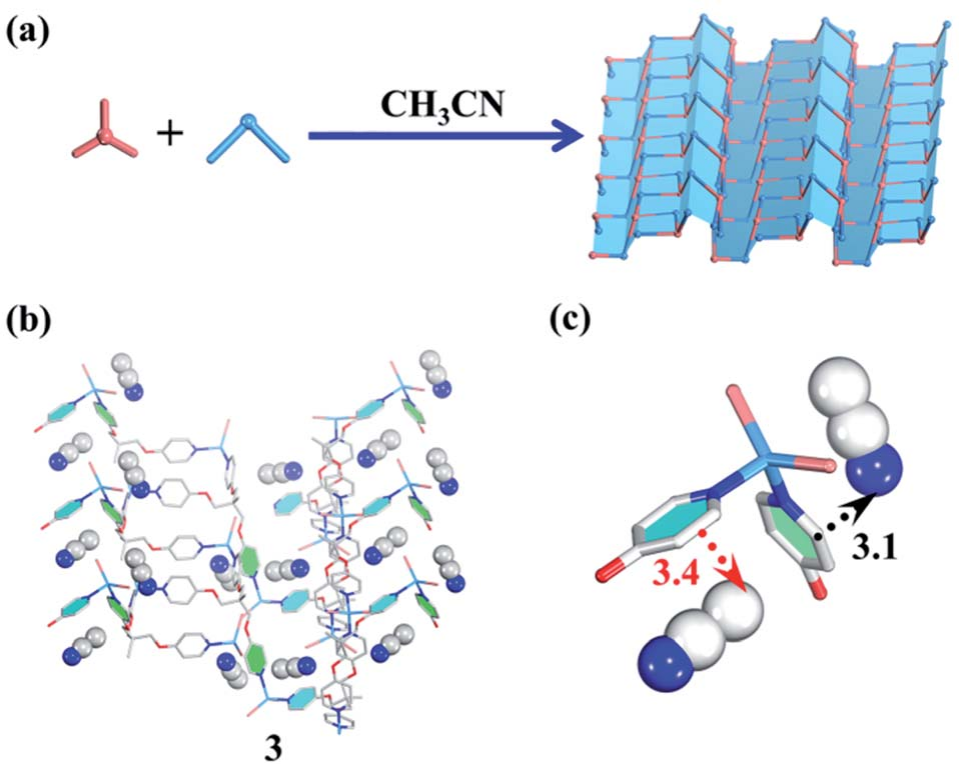
(a) Guest-driven assembly of the 2D layered metal–organic assembly. (b) The structure of **3**. (b and c) The template effect of CH_3_CN (blue/white balls represent N/C atoms respectively).

**Fig. 4:**
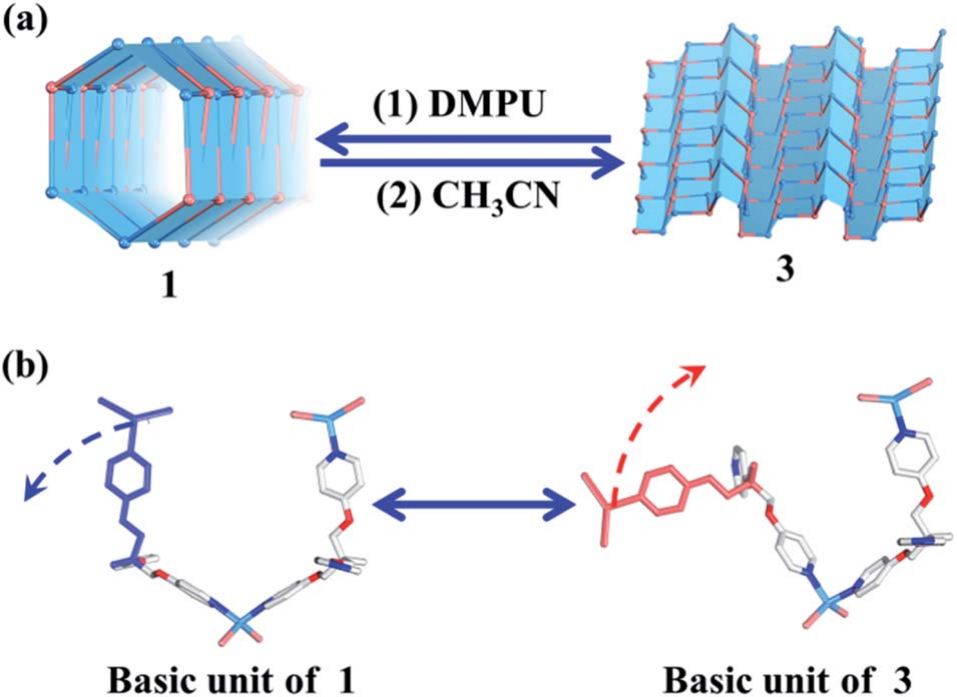
(a) Reversible tube/sheet transformations between **1** and **3**. (b) The transformation mechanism based on the asymmetric units of **1** and **3**.

**Fig. 5:**
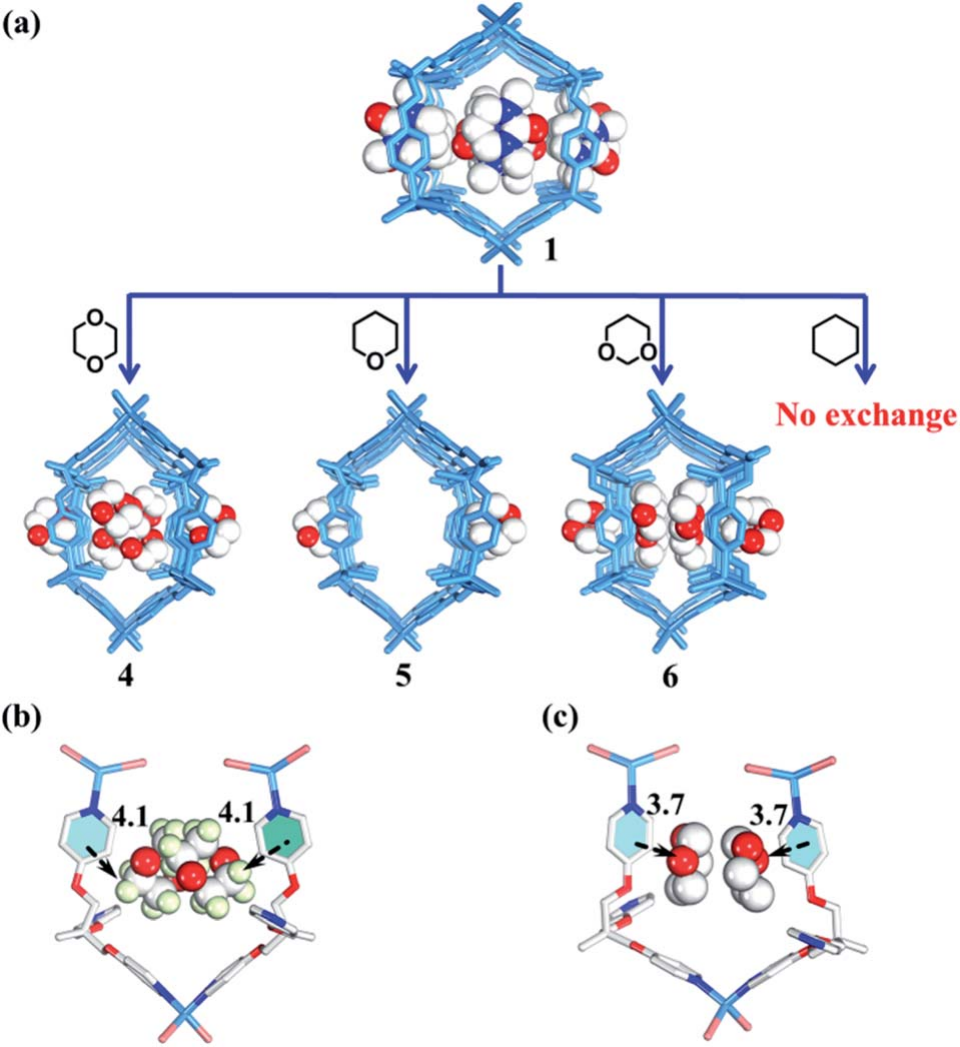
(a) Selective encapsulation of guest molecules with a similar size but different polarity by nano-channels. (b and c) Interactions between guest molecules and nano-channels (white/red/pale green balls represent C/O/H atoms respectively).

**Fig. 6:**
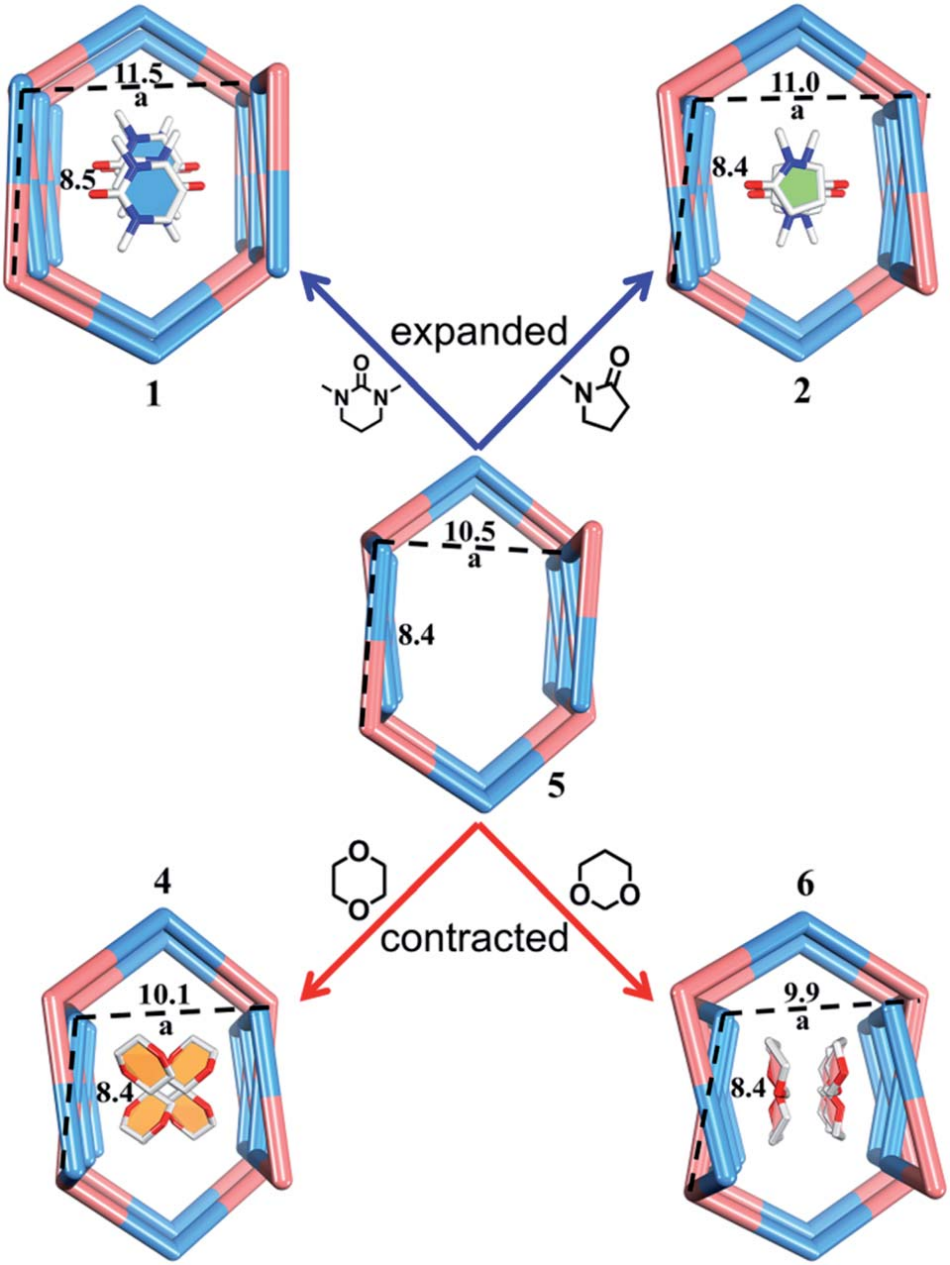
Guest-dependent deformations of the nano-channel.

**Fig. 7:**
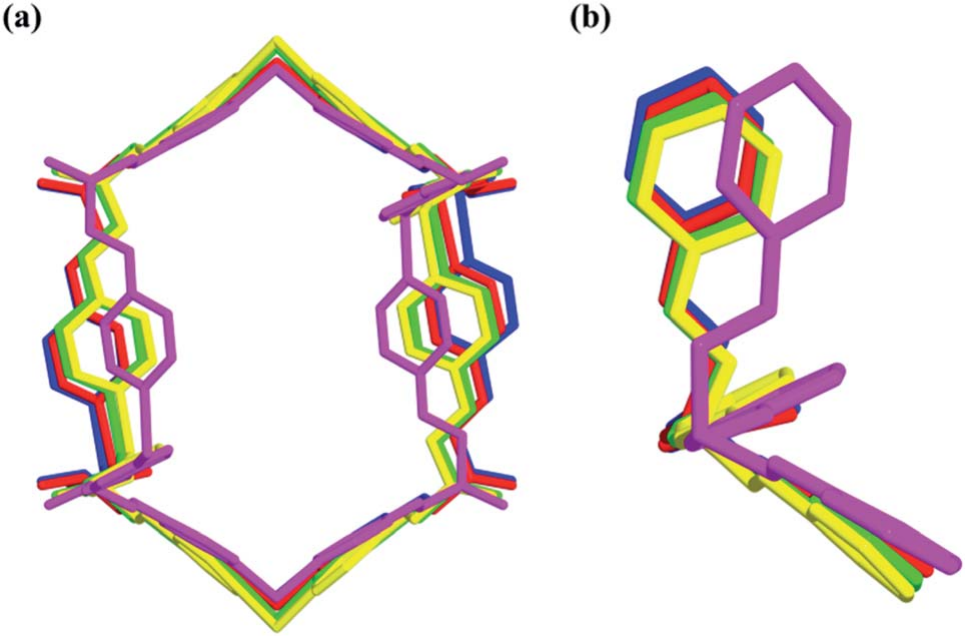
Nano-channels and conformations of TPOME ligands in different compounds (blue/red/green/yellow/violet represent **1**, **2**, **4**, **5**, and **6**, respectively).

**Fig. 8:**
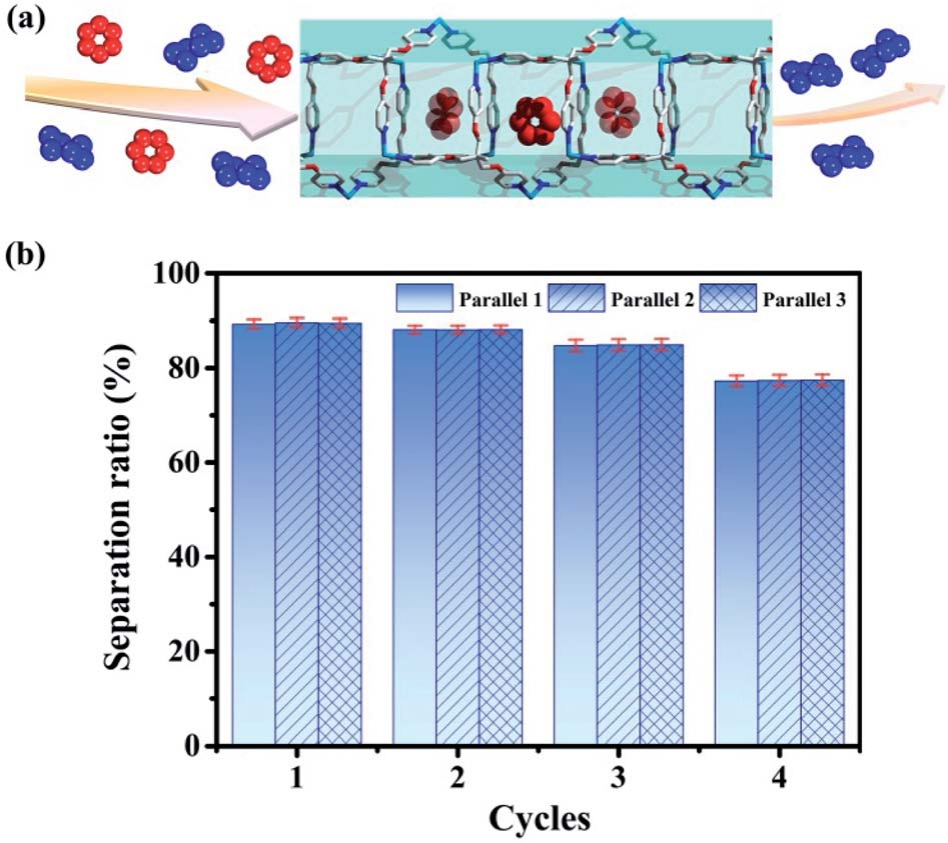
(a) Separation of benzene/cyclohexane (red represents benzene and blue represents cyclohexane) by tube **1**. (b) Separation ratios in 4 cycles (the values are determined by GC after adsorption).

Both carbon nanotubes and graphene are constructed from sp^2^ hybridized carbon atoms. We thus conceive that both the 1D tubular assembly and the corresponding 2D layered assembly could be assembled from suitable building blocks with *C*_3_ symmetry. Thus, we select flexible ligands with *C*_3_ symmetry as donors and two-coordinated metal ions as acceptors for the self-assembly of both adaptive 1D metal–organic tubes and the corresponding 2D layered metal–organic assembly (Fig. 1). In order to prepare desired assemblies, M–N (M = Pd(ii), Ag(i), Zn(ii), N = pyridyl ligand) units with moderate coordination strength have been selected and tested, then a series of flexible ligands has been synthesized,^68,69^ and various two-coordinated metal acceptors such as orthogonal ethylenediamine “capping” Pd(ii), linear Ag(i) salts, and rhombic ZnX_2_ (X = Cl^−^, Br^−^, I^−^) have been screened in the trials. We finally find that the combination of tri-((pyridin-4-yloxy)methyl)ethane (TPOME) and ZnBr_2_ leads to the successful assembly of both 1D metal–organic tubes with nano-channels and 2D layered metal–organic assembly driven by different templates (Fig. 1–3). Directly rolling-up the 2D sheet structure into the corresponding 1D tubular structure and unzipping the 1D tubular structure into the corresponding 2D sheet structure can be realized smoothly in our assembly system, and the key intermediate accompanied by such a reversible sheet-to-tube process has been identified (Fig. 4). Remarkably, the newly formed nano-channel displays polarity-selectivity guest encapsulation behavior, and can be expanded or contracted through guest-driven adaptive deformation. Moreover, even though guest molecules have similar sizes, such guest-driven adaptive deformations can also be obviously distinguished by X-ray snap-shot analysis and powder X-ray diffraction (PXRD) in detail (Fig. 5–7). Finally, efficient separation of key chemicals benzene/hexane with similar sizes and boiling points has been realized *via* polarity-selectivity and adaptive deformation of nano-channels (Fig. 8).

## Results and discussion

### Self-assembly of 1D metal–organic tubes with nano-channels driven by circular templates

Slow diffusion of Et_2_O vapor into a 1,3-dimethyl-tetrahydropyrimidin-2(1*H*)-one (DMPU) solution of the TPOME ligand and ZnBr_2_ leads to colorless rod-like crystals of assembly **1**, formulated as [(Zn_3_L_2_Br_6_)·(C_6_H_12_N_2_O)_4_]_*n*_ (Table S1†). Single crystal X-ray diffraction analysis reveals that its asymmetric unit contains three Zn(ii) ions, two TPOME ligands, six Br^−^ anions, and four dissociative DMPU molecules (Fig. S1†). Each Zn(ii) ion has a slightly distorted tetrahedral geometry through coordinating with two bromide anions and two N atoms from two distinct ligands. The ZnBr_2_ units link TPOME ligands to form an infinite 1D tubular structure with a dimension of 11.5 × 8.5 Å nano-channels (Fig. 2b, c and 6). Some of the DMPU molecules stack closely along the 1D nano-channel of tube **1** with a 4.2 Å distance, and other DMPU molecules fill in the windows of nano-channels (Fig. 2c). By simplifying TPOME ligands as 3-connecting nodes, assembly **1** has a typical (4, 3) ladder-shaped chain (Fig. S7†). We speculate that the DMPU molecules may serve as circular templates, which induce the TPOME ligands to self-assemble into a circular tube (Fig. 2d). In order to address the growth mechanism, we replace Et_2_O with water for crystal growth, which gives the same assembly **1**, suggesting that Et_2_O is not the necessary template. Furthermore, using another circular molecule *N*-methyl pyrrolidone (NMP) results in another similar tube **2**, formulated as [(Zn_3_L_2_Br_6_)·(C_5_H_9_N_2_O)_4_]_*n*_ (Table S1, Fig. S2†). Driven by the smaller NMP template, the nano-channel of **2** slightly shrinks (11.0 × 8.4 Å) (Fig. 2e, f and 6). This result not only proves that the circular DMPU/NMP molecules can serve as templates for the rational construction of metal–organic tubes, but also demonstrates that the size of the nano-channel is adaptive and can be tuned by different templates (Fig. 2a). Although compounds **1** and **2** contain a discrete nanotubular structure, nanometer-scale tubular units are not observed in scanning electron microscopy (SEM) due to their excellent stability (Fig. S11†).^23,31^

### Self-assembly of the 2D layered metal–organic assembly driven by non-circular templates

In terms of structural diversity, the raised question is whether the corresponding 2D sheet structure can also be assembled from the same building units. After many attempts, we finally found that the use of CH_3_CN as a template and *N*,*N*-diethylformamide (DEF) as a solvent can lead to the 2D layered metal–organic assembly (Fig. 3a). Slowly diffusing CH_3_CN vapor into a DEF solution of the TPOME ligand and ZnBr_2_ gives colorless block crystals of assembly **3**, formulated as [(Zn_3_L_2_Br_6_)·(CH_3_CN)_2_]_*n*_ (Table S1†). Single crystal X-ray diffraction analysis reveals that **3** crystallizes in the space group *Pbca*, and its asymmetric unit contains three Zn(ii) ions, two TPOME ligands, six Br^−^ anions, and two associative CH_3_CN molecules (Fig. S3†). Each Zn(ii) ion also has a slightly distorted tetrahedral geometry as **1**. In sharp contrast to **1**, herein, ZnBr_2_ units link TPOME ligands to form an infinite 2D wavy sheet structure with 2-fold interpenetration (Fig. 3b and S8†), in which CH_3_CN molecules distribute parallel to the pyridine rings with strong CH–π and C≡N–π interactions (Fig. 3b and c). By simplifying TPOME ligands as 3-connecting nodes, the resulted 2D wavy sheet has a typical (6, 3) net, the same as graphene (Fig. S9†). Herein, the CH_3_CN molecules should serve as templates that induce the TPOME ligands to self-assemble into an infinite 2D sheet structure (Fig. 3b and c). To gain further insight into the effect of CH_3_CN and DEF, we first used other solvents (H_2_O/Et_2_O/EtOH) to replace CH_3_CN; however, no similar 2D sheet structure could be obtained. Furthermore, diffusing CH_3_CN vapor into the DMPU/NMP solution also could not lead to the formation of a similar 2D sheet structure. These results demonstrate that the 2D sheet structure of assembly **3** results from the synergic effect of CH_3_CN templates and DEF solvent. Although compound **3** contains a 2D sheet structure, nanometer-scale metal–organic sheets are not observed in SEM (Fig. S11†).^70^

### Reversible sheet-to-tube transformation

Without considering the guest molecules, **1** and **3** are a pair of isomers. Topological analysis also suggests that the reversible transformation of the (4, 3) chain into the (6, 3) net only requires simple deconstruction and reconstruction (Fig. S10†). As such, the reversible transformation between **1** and **3** should be feasible. After many attempts, we find that soaking **3** (2D layered metal–organic assembly) in the DMPU solution under the atmosphere can directly lead to compound **1** (1D metal–organic tube). However, reconstruction of **1** into **3** needs two steps, *i.e.*, dissolving **1** in DEF under ultrasonic treatment firstly; then slowly diffusing CH_3_CN vapor into such solution to form corresponding **3**. The reversible sheet-to-tube conversion should be a dynamic assembly process which contains deconstruction of a given infinite structure into basic units and reconstruction of such newly formed basic units into another infinite structure driven by corresponding templates. Specifically, when rolling-up the 2D sheet structure of **3** into the corresponding 1D tubular structure of **1**, **3** should be slowly dissolved in the DMPU solution to form basic building units comprised of the TPOME ligand and ZnBr_2_, which re-assemble into **1** driven by DMPU templates and promoted by the water vapor of the atmosphere. Unzipping the 1D tubular structure of **1** into its corresponding 2D sheet structure of **3** also follows a similar process. This result confirms that the reversible tube-to-sheet conversion can be carried out smoothly in our assembly system (Fig. 4a). In order to gain further insight into the transformation intermediate in the solution state, ^1^H NMR and high-resolution-mass spectrometry (HR-MS) have been applied during both tube-to-sheet and sheet-to-tube conversions. ^1^H NMR spectra show a clear down-field shift for signals of the TPOME ligand, especially for H_A_ and H_B_ on the pyridine units (Fig. S12†), suggesting that a dynamic coordination library is formed. Furthermore, the same [Zn_3_L_2_Br_5_]^+^ (*m*/*z* 1298.68) species has been observed during both transformation processes (Fig. S13†). Therefore, we propose that the key intermediate during the reversible sheet-to-tube conversion should be a dynamic Zn_3_L_2_ unit. As shown in Fig. 4b, the asymmetric units of **1** and **3** are also a pair of Zn_3_L_2_ isomers, which can be reversibly transformed by simple rotation of one chelation arm on the TPOME ligand.

### Selective encapsulation of guest molecules with a similar size but different polarity

Rational design of adaptive cavities like pockets of enzymes remains a big challenge, which is attributed to the lack of an in-depth understanding about the nature of the interactions between adaptive host and guest molecules.^1,65^ As mentioned above, the nano-channel of **1** is adaptive. We speculate that such adaptive nano-channels may distinguish similar substrates like enzymes. 1,4-Dioxane and tetrahydropyrane (THP) with a similar size but different polarity are firstly selected to test the binding properties of the nano-channel. Soaking the single crystals of **1** into 1,4-dioxane and THP leads to encapsulated crystal **4** {[(Zn_3_L_2_Br_6_)·(C_4_H_8_O_2_)_4_]_*n*_} and **5** {[(Zn_3_L_2_Br_6_)·(C_5_H_10_O)_2_]_*n*_} (Table S2†), correspondingly, through a single-crystal-to-single-crystal transformation *via* solvent exchange (Fig. 5a). Single crystal X-ray diffraction analyses demonstrate that both dioxane and THP can replace DMPU molecules within **1***via* solvent exchange. However, their distribution and effect on nano-channels are significantly different: 1,4-dioxane molecules with stronger polarity can stack orderly along the nano-channel while less polar THP cannot. These results indicate that the microenvironment within the nano-channel is polar. In order to validate such a presumption, another two guest molecules, *i.e.*, 1,3-dioxane and cyclohexane were tested. Note that 1,3-dioxane has a slightly higher polarity than 1,4-dioxane, while cyclohexane displays the weakest polarity among all the tested molecules. Indeed, 1,3-dioxane with higher polarity can also stack orderly along the nano-channel of **6** {[(Zn_3_L_2_Br_6_)·(C_4_H_8_O_2_)_4_]_*n*_} (Table S2†), similarly to 1,4-dioxane in **4**, but more compact packing of 1,3-dioxane than 1,4-dioxane makes the nano-channel of **6** (9.9 × 8.4 Å) adopt a smaller diameter than that of **4** (Fig. 5c and 6). Cyclohexane with the lowest polarity cannot enter the channel of **1** even after several weeks of soaking. As such, we confirm that the microenvironment of assembled nano-channels is polar, and only polar guest molecules can be selectively encapsulated.

### Observation of adaptive deformation of nano-channels by single crystal X-ray diffraction

Compared to the guest-free nano-channel of **5** (10.5 × 8.4 Å) (Fig. 6), different guest molecules in **1**, **2**, **4**, and **6** lead to the adaptive deformation of their corresponding nano-channels, as confirmed in detail by X-ray crystallography.^71,72^ Specifically, the nano-channels of **1** and **2** are expanded by DMPU and NMP molecules, and the nano-channels of **4** and **6** are contracted by 1,4-dioxane and 1,3-dioxane molecules (Fig. 7a); and their sizes are arranged as follows: **6** < **4** < **5** < **2** < **1**.

Such guest-driven nano-channel changes can also be detected by PXRD analyses. Compounds **1**, **5**, **4**, and **6** belong to the same space group *Pna*2_1_; in their XRD patterns, all of their [002] Bragg reflections correspond to the interlayer spacing of nano-channels along the *c* axis (Fig. S14–S19†). As shown in Fig. S20,† the 2*θ* of the [002] reflection of compounds **1**, **5**, **4**, and **6** is about 4.98°, 5.24°, 5.64°, and 5.79°, respectively; and the corresponding order is **1** < **5** < **4** < **6**. According to Bragg's law (2*d* sin *θ* = *nλ*), the distances (*d*_002_) of [002] reflections of the samples **1**, **4**, **5**, and **6** should vary inversely with the value of 2*θ*, demonstrating that the *a* values of nano-channels of samples **1**, **4**, **5**, and **6** are arranged in an opposite trend: **1** > **5** > **4** > **6**. This tendency is consistent with the result from single X-ray diffraction analysis of a single crystal (Fig. S19†). Both single X-ray diffraction analyses and PXRD analyses demonstrate that the nano-channel can be expanded or contracted by different guest molecules. The adaptive deformation associated with the guest molecules is fully reversible; after soaking samples **4**, **5**, and **6** into the DMPU solution for three days, the frameworks can be recovered the same as that of tube **1** (Fig. S21†).

In short, reversible guest-driven adaptive deformation of nano-channels has been realized, and the possible mechanism of adaptive deformation may be described as the following. The polar microenvironment of nano-channels will help the polar guest molecules stack orderly. In a confinement cavity, the host–guest interaction between adsorbed molecules and nano-channels will be improved, such amplified host–guest interaction will change the shape of the nano-channel through a slight conformational change of the TPOME ligand (Fig. 7b). In particular, larger DMPU/NMP molecules can expand the free nano-channel because of their larger steric hindrance; while smaller dioxane molecules will shrink the nano-channel due to the presence of strong dipole–dipole interactions between guest molecules and nano-channels (Fig. 5b and c). Generally, introduction of extra guest molecules into an empty cavity will expand its size; guest-driven contraction of the coordination cavity is a highly rare phenomenon. Moreover, for nano-channels of tube **1**, even though guest molecules have similar sizes, such guest-driven adaptive deformations can also be obviously distinguished by X-ray snap-shot analysis in detail. As far as we know, many flexible metal–organic frameworks (MOFs) can also be deformed by various external stimuli, including exchange and loss of solvent, temperature change, and pressure adjustment.^73–89^ However, flexible MOFs like tube **1** that can accurately distinguish very similar molecules and produce adaptive deformation is rarely reported.^90–92^ Such unusual deformation comprised of tube **1** not only advances our understanding of the interactions between adaptive host and guest molecules,^93^ but also provides a new strategy for developing new porous materials for the separation of isomers or homologs with similar sizes.

### Effective separation of benzene and cyclohexane

Separation of cyclohexane and benzene has been a most challenging process for the petrochemical industry due to their similar sizes and boiling points. The traditional distillation process for the separation of cyclohexane and benzene requires complex equipment and high energy consumption.^94,95^ It will be highly economical to directly separate benzene and cyclohexane in the liquid phase. As mentioned above, tube **1** prefers to adsorb the molecules with stronger polarity and cyclohexane cannot be adsorbed, so using such dynamic tubes to selectively separate benzene and cyclohexane which have a similar size but different polarity seems possible; ideally, benzene with stronger polarity should be preferably adsorbed by tube **1**. Before separation experiments, we should firstly confirm that tube **1** can adsorb and release benzene reversibly. Therefore, we hope to calculate the pore size and pore volume of tube **1** by N_2_ adsorption experiments; however, the framework of tube **1** is broken after vacuum activation (Fig. S22†). So we directly carried out the liquid phase adsorption experiment, and soaking the single crystals of tube **1** into benzene solution three days leads to a benzene-encapsulated sample (compound **7**). Unfortunately, high quality single crystals of compound **7** suitable for single crystal X-ray diffraction cannot be obtained. So various testing methods including ^1^H NMR, thermogravimetric (TG) analysis, elemental analysis, and infrared spectroscopy (IR) were used to confirm the composition of compound **7**. In order to determine the content of benzene in compound **7**, we directly dissolved compound **7** in DMSO-D_6_ solution. ^1^H NMR data demonstrate that the ratio of ligand to benzene is 2 : 2.5; at the same time, no peaks corresponding to DMPU molecules have been observed, which means that the DMPU molecules have been completely exchanged by benzene molecules (Fig. S23†). So the molecular formula of compound **7** is calculated to be {[(Zn_3_L_2_Br_6_)·(C_6_H_6_)_2.5_]_*n*_}. This can be further confirmed by the data of TG, IR, and elemental analysis. IR spectra further prove that compound **7** does not contain DMPU molecules (Fig. S27†). Moreover, 12.4% of weight loss of compound **7** is in good agreement with its molecular formula (Fig. S24†), and the measured and theoretical values of elemental analysis can also be well matched. All data confirm that the molecular formula of compound **7** is indeed {[(Zn_3_L_2_Br_6_)·(C_6_H_6_)_2.5_]_*n*_}, suggesting that the saturated adsorption capacity of tube **1** for benzene is about 142 mg g^−1^. After confirming that tube **1** can adsorb benzene, we need to further confirm that tube **1** does not decompose after adsorbing benzene and the adsorbed benzene molecules can be released. So we use PXRD, IR, SEM, Transmission Electron Microscopy (TEM), and elemental mapping techniques to *in situ* detect the conversion of tube **1** and compound **7**. When tube **1** is converted to compound **7** through benzene exchange, the relatively strong and clean PXRD patterns of compound **7** indicate that compound **7** is a crystalline material like compound **4** rather than amorphous powder (Fig. S25†). Both SEM and TEM data show that the size of compound **7** is similar to that of tube **1**, which means that the benzene exchange does not break the block tube **1** into fragments (Fig. S28–S30†); compared with tube **1**, there are a few cracks on the surface of compound **7**, which may cause compound **7** to be unsuitable for single crystal X-ray diffraction. Elemental mapping data show that the distribution of C, N, O, Zn, and Br in compound **7** is as uniform as that of tube **1** (Fig. S29 and S30†), meaning that no new impurities are produced by benzene exchange. All these data mean that benzene adsorption will not decompose the framework of tube **1**. When soaking compound **7** in a small amount of DMPU solution for 24 hours, all data including PXRD patterns, IR spectra, SEM, TEM, and elemental mapping prove that compound **7** can be transformed into tube **1**. Notably, when compound **7** is restored to tube **1**, not only will the framework be restored (Fig. S26†), the cracks on the surface can also be repaired (Fig. S28–S31†), and the adsorbed benzene molecules will be completely released (Fig. S27†). In a word, benzene can be encapsulated into the nano-channels of tube **1**, and the saturated adsorption of benzene enables tube **1** to *in situ* transform into compound **7**, which is a benzene-induced deformation product rather than a decomposition product destroyed by benzene. The benzene molecules adsorbed in the nano-channels of compound **7** can be released through DMPU exchange, accompanied by the restoration of the framework of tube **1**. Therefore, tube **1** can be used to adsorb and release benzene molecules reversibly *via* solvent exchange.

Then we use tube **1** to separate benzene and cyclohexane as follows: firstly soaking crystals of nanotube **1** into the mixed solution of benzene and cyclohexane (1 : 1) for 12 hours; then using chloroform to extract the adsorbed analyte for the gas chromatography test (Agilent 7890A with DB-WAXETR column). Indeed, tube **1** can selectively adsorb 89.6% of benzene from the mixed solution of benzene and cyclohexane (Fig. 8, S34–S36, S40 and S41, Table S5†), after re-soaking the used-sample into a small amount of DMPU solution, and the framework can be recovered (Fig. S32†) and reused for another two cycles without reduction of separation efficiency (88.1%, 84.7%) (Fig. 8, S37 and S38, Table S5†). In the fourth cycle of separation, its selectivity is slightly reduced to 77.4% (Fig. 8 and S39, Table S5†). This is very confusing, because the PXRD data show that even after four cycles of separation, the framework of tube **1** does not collapse (Fig. S32†). In order to extract the analyte from tube **1** for testing as soon as possible, we perform 30 minutes of ultrasonic operation on the benzene-adsorbed tube **1** each time, such long-term ultrasonic operation may break the tube **1** block into fragments. If the sample block is broken, the length of nano-channels in the sample fragments will become significantly shorter than that of the bulk, so that the analytes cannot be perfectly stacked as before in the block, which may reduce its separation efficiency. Then we performed SEM tests on the samples of tube **1** before and after separation; SEM data demonstrate that the size of tube **1** after 4 cycles of separation indeed significantly decreases (Fig. S33†). Considering that the framework of tube **1** remains unchanged (Fig. S32†), we believe that the reduction in separation efficiency is due to the fact that tube **1** changed from block to fragments (Fig. S33†). In order to restore its separation efficiency, we need to restore the size of used tube **1** through regeneration. Fortunately, with help of ultrasonic treatment, the used sample of tube **1** can be regenerated readily by recrystallization in DMPU solution. Regeneration of used tube **1** into fresh one without losing efficiency is expected to reduce the cost of separation. This result not only enriches the applications of metal–organic tubes but also provides a potential and practical method for the separation of benzene and cyclohexane. As described previously, the industrial separation of benzene and cyclohexane is achieved by rectifying their mixed steam. The selective adsorption based on porous adsorbents can improve the separation efficiency and reduce energy consumption. Then many MOF materials have been used to selectively separate benzene and cyclohexane.^96–105^ However, the separation experiments of benzene and cyclohexane based on such reported MOFs are also achieved through adsorption of steam. This means that it is still necessary to turn benzene and cyclohexane into steam by heating, which cannot completely solve the problem of energy consumption. Compared with steam separation, liquid phase separation using tube **1** will be more energy-efficient.

## Conclusions

In summary, a new strategy for the rational construction of metal–organic tubes with nano-channels has been developed, which allows reversible sheet-to-tube transformation in the metal–organic tube area to be carried out in detail. Furthermore, the newly formed nano-channel displays excellent polarity-selectivity for encapsulation of different guest molecules, and can be further expanded or contracted through guest-driven adaptive deformation; even induced by guest molecules with similar sizes, the adaptive deformations can also be obviously distinguished by X-ray snap-shot analysis. Finally, the key chemicals benzene/hexane with similar sizes can also be effectively separated by such nano-channels in the liquid phase. Our work not only provides a new synthetic strategy for the rational synthesis of metal–organic tubes, but also gives a potential method for the construction of adaptive host-like enzymes and an in-depth understanding of the nature of adaptive host and guest molecules.

## Conflicts of interest

There are no conflicts to declare.

## Supplementary Material

SC-011-D0SC01176B-s001

SC-011-D0SC01176B-s002
