# Supplementary material for: Metal–organic tube or layered assembly: reversible sheet-to-tube transformation and adaptive recognition
Source: Chem Sci. 2020 Aug 12;11(36):9818–26. doi: 10.1039/d0sc01176b (PMC8162108; doi:10.1039/d0sc01176b)
Supplement: SC-011-D0SC01176B-s001 [file SC-011-D0SC01176B-s001.pdf]

Electronic Supplementary Material (ESI) for Chemical Science.
This journal is © The Royal Society of Chemistry 2020

Supporting information

Experimental methods

General: All of reagents were commercially available and used as purchased. C, H, and N microanalyses were measured using an elemental Vairo EL analyzer, ^{1}HNMR and ^{13}CNMR spectra were recorded at Bruker AM-400 (400 MHZ) spectrometer.

Synthesis of L

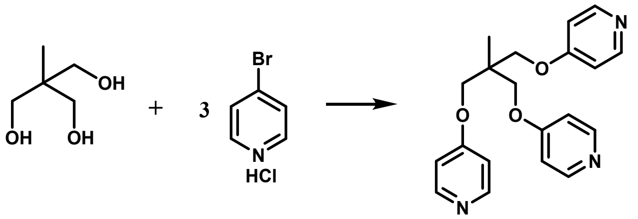

A mixture of 1,1,1-Tris(Hydroxymethyl)ethane (1.202 g, 10 mmol), 4-Bromopyridine Hydrochloride (5.834 g, 30 mmol), KOH(6 g) were stirred in 160 mL DMSO at 60℃, after 3 days stirring, the solution was filtered, and the solvent was extracted with $CHCl_3/H_2O$,and the solution was evaporated to dryness at Rotary Evaporator. The residue was purified by column chromatography on silica gel with MeOH /$CHCl_3$ (1:10). The product was obtained as a white powder (1.90 g, yield: 54.13%). ^{1}H NMR (400 MHz, DMSO) δ: 8.36 (6H, d, *J*= 6), 6.99 (6H, d, *J*= m), 4.17 (6H, s), 1.25 (3H, 3). ^{13}C NMR (400 MHz, DMSO) δ: 164.78, 151.41, 110.75, 69.40, 39.99, 17.24. ESI-MS (m/z): 352 ($M+H^+$). Anal. Calcd for $C_{20}H_{21}N_3O_3$ (%): C, 68.36; H, 6.02; N, 11.96; Found (%): C, 68.51; H, 6.01; N, 12.04. IR (KBr) ν: 3033, 2980, 2945, 2870, 1590, 1570, 1503, 1467, 1457, 1418, 1284, 1208, 1047, 998, 847, 814, 539, 518 cm^{-1}.

Synthesis of compound 1

Sonicating of TPOME ligand (7 mg, 0.02 mmol) with $ZnBr_2$ (6.8 mg, 0.03 mmol) in 1ml 1,3-Dimethyl-3,4,5,6-tetrahydro-2(1H)-pyrimidinone (DMPU) solution 5 minutes, after that, slowly diffusing Et_2O/H_2O vapor into such solution five days to give

colorless block crystals of compound **1** (14 mg, yield:74%). Anal. Calcd for $C_{64}H_{90}Br_6N_{14}O_{10}Zn_3$ (%): C, 40.61; H, 4.76; N, 10.36; Found (%): C, 40.19; H, 5.1; N, 10.77.

Synthesis of compound 2

Sonicating of TPOME ligand (7 mg, 0.02 mmol) with $ZnBr_2$ (6.8 mg, 0.03 mmol) in 1ml N-Methyl pyrrolidone (NMP) solution 5 minutes, after that, slowly diffusing Et_2O/H_2O vapor into such solution five days to give colorless block crystals of compound **2** (14 mg, yield: 74%). Anal. Calcd for $C_{60}H_{78}N_{10}O_{10}Zn_3Br_6$ (%): C, 40.56; H, 4.39; N, 7.89; Found (%): C, 40.04; H, 4.51; N, 7.64.

Synthesis of compound 3

Sonicating of TPOME ligand (7 mg, 0.02 mmol) with $ZnBr_2$ (6.8 mg, 0.03 mmol) in 1ml diethylformamide (DEF) solution 5 minutes, after that, slowly diffusing CH_3CN vapor into such solution ten days to give colorless block rod crystals of compound **3** (11 mg, yield: 75%). Anal. Calcd for $C_{44}H_{48}Br_6N_8O_6Zn_3$ (%): C, 36.15; H, 3.29; N, 7.67; Found (%): C, 36.04; H, 3.7; N, 7.45.

Sheet-to-tube Transformation:

Directly soaking crystals of compound **3** in the DMPU solution two months lead to crystals of compound **1**.

Tube-to-sheet Transformation:

Sonicating of crystals of compound **1** in 1 ml DEF solution 5 minutes, after that, slowly diffusing CH_3CN vapor into such solution two weeks give colorless block crystals of compound **3.**

Encapsulating 1, 4-dioxane molecules (Synthesis of compound 4)

Immersing crystals of compound **1** in solution of 1, 4-dioxane 3 days leads to the

crystals of compound **4**. Anal. Calcd for $C_{56}H_{74}N_6O_{14}Zn_3Br_6$ (%): C, 38.83; H, 4.28; N, 4.85; Found (%): C, 38.45; H, 4.43; N, 5.01.

Encapsulating tetrahydropyrane molecules (Synthesis of compound5)

Immersing crystals of compound **1** in solution of tetrahydropyrane 3 days leads to the crystals of compound **5**. Anal. Calcd for $C_{50}H_{62}Br_6N_6O_8Zn_3$ (%): C, 38.69; H, 4.0; N, 5.42; Found (%): C, 38.31; H, 4.19; N, 5.61.

Encapsulating 1, 3-dioxane molecules (Synthesis of compound 6)

Immersing crystals of compound **1** in solution of 1, 3-dioxane 3 days leads to the crystals of compound **6**. Anal. Calcd for $C_{56}H_{74}Br_6N_6O_{14}Zn_3$ (%): C, 38.83; H, 4.28; N, 4.85; Found (%): C, 38.43; H, 4.55; N, 5.15.

Encapsulating benzene molecules (Synthesis of compound 7)

Immersing crystals of compound **1** in solution of benzene 3 days leads to the crystals of compound **7**. Anal. Calcd for $C_{55}H_{57}Br_6N_6O_6Zn_3$ (%): C, 41.94; H, 3.62; N, 5.34; Found (%): C, 41.59; H, 4.01; N, 5.58.

Recovering compounds **4**, **5**, **6**, **7** into compound **1**

Immersing crystals of compounds **4**, **5**, **6**, **7** in a small amount of DMPU solution 3 days leads to the crystals of compound **1**.

Separation of cyclohexane and benzene

20 mg of compound **1** was soaked in a 1:1 (vol/vol) mixture of cyclohexane (2 mL) and benzene (2 mL) for 10 hours at room temperature. The soaked crystal was collected on filter paper, then washed with a small amount of chloroform three times. Finally, using chloroform solution to extract such encapsulated analyte from channels of crystal with help by ultrasound operation 30 minutes. The composition of encapsulated analyte was determined by GC (Agilent 7890A) with DB-WAXETR column.

X-ray crystallography

Data collections were all performed on a Mercury CCD diffractometer with graphite monochromated Mo Ka radiation (λ = 0.71073 Å). The structures were solved by direct methods, and all calculations were performed using the SHELXL package[1]. The structures 1–6 were refined by full matrix least-squares with anisotropic displacement parameters for non-hydrogen atoms. All hydrogen atoms were generated geometrically and treated as riding. We employed PLATON/SQUEEZE[2-3] to calculate the contribution to the diffraction from the solvent region and thereby produced a set of solvent-free diffraction intensities. The final formula was calculated from the SQUEEZE[2-3] results. The crystallographic data are summarized in Table S1 and Table S2. CCDC 1919898-99, 1919901-03, 1919906 contain the supplementary crystallographic data for 1-6. These data can be obtained free of charge from The Cambridge Crystallographic Data Centre via www.ccdc.cam.ac.uk/data_request/cif.

Table S1. Crystal data and structure determination summary for **1–3.**

Compounds	**1**	**2**	**3**
CCDC deposit no.	1919898	1919899	1919901
Formula	$C_{64}H_{90}Br_6N_{14}O_{10}Zn_3$	$C_{60}H_{78}N_{10}O_{10}Zn_3Br_6$	$C_{44}H_{48}Br_6N_8O_6Zn_3$
Formula weight	1891.06	1774.89	1460.47
Crystal system	orthorhombic	orthorhombic	orthorhombic
Space group	$Pna2_1$	*Pbcn*	*Pbca*
a (Å)	16.5576(6)	33.1022(15)	15.4998(6)
b (Å)	13.6347(6)	13.6273(4)	25.3392(11)
c (Å)	35.0720(16)	16.0057(5)	27.2058(8)
α (°)	90	90	90
β (°)	90	90	90
γ (°)	90	90	90
V (Å^3)	7917.8(6)	7220.1(5)	10685.1(7)

Z	4	4	8
D_c (g/cm^{-3})	1.586	1.633	1.816
μ (mm^{-1})	3.990	5.534	5.875
F(000)	3808.0	3524.7	5728
θ range (°)	2.257, 25.027	3.51, 73.14	2.762, 26.373
No. of unique reflections / collected	15194/55963	7060/17519	10899/60857
Goodness-of-fit (GOOF)	1.043	1.170	1.052
Final R indices ($I > 2\sigma(I)$)	R_1 = 0.0539 ωR_2 = 0.1291	R_1 = 0.0659 ωR_2 = 0.1295	R_1 = 0.0708 ωR_2 = 0.1452

Table S2. Crystal data and structure determination summary for **4**–**6**.

Compounds	**4**	**5**	**6**
CCDC deposit no.	1919902	1919903	1919906
Formula	$C_{56}H_{74}N_6O_{14}Zn_3Br_6$	$C_{50}H_{62}Br_6N_6O_8Zn_3$	$C_{56}H_{74}Br_6N_6O_{14}Zn_3$
Formula weight	1730.78	1550.62	1730.78
Crystal system	orthorhombic	orthorhombic	orthorhombic
Space group	$Pna2_1$	$Pna2_1$	$Pna2_1$
a (Å)	15.6587(3)	15.889(2)	16.3631(3)
b (Å)	13.6267(18)	13.4934(13)	13.1544(2)
c (Å)	32.0101(2)	33.290(8)	30.7790(5)
α (°)	90	90	90
β (°)	90	90	90
γ (°)	90	90	90
V (Å^3)	6830.2(18)	7137(2)	6625.08(19)
Z	4	4	4
D_c (g/cm^{-3})	1.683	1.443	1.735
μ (mm^{-1})	3.929	3.675	4.051
F (000)	3456.0	3072.0	3456.0

θ range (°)	2.4, 52.928	2.308, 56.907	2.496, 56.909
No. of unique reflections / collected	11948/230666	14560/252053	13525/246534
Goodness-of-fit (GOOF)	1.088	1.087	1.038
Final R indices ($I > 2\sigma(I)$)	$R_1 = 0.1275$<br>$\omega R_2 = 0.2570$	$R_1 = 0.1125$<br>$\omega R_2 = 0.2757$	$R_1 = 0.1073$<br>$\omega R_2 = 0.2277$

Table S3. The parameters of structural refinment for **1** and **1** without guest molecules.

Compounds	Space group	R_1	ωR_2	Flack
**1**	$Pna2_1$	0.0539	0.1386	0.5
**1-squeeze**	$Pna2_1$	0.0522	0.1314	0.492

Table S4. The parameters of structural refinment with different space group for **4-6**.

Compounds	Space group	R_1	ωR_2	Flack
**4**	*Pbcn*	0.1999	0.4088	
	$Pna2_1$	0.1275	0.2570	0.51
**5**	*Pbcn*	0.1477	0.2731	
	$Pna2_1$	0.1125	0.2757	0.48
**6**	*Pbcn*	0.1651	0.4015	
	$Pna2_1$	0.1073	0.2277	0.499

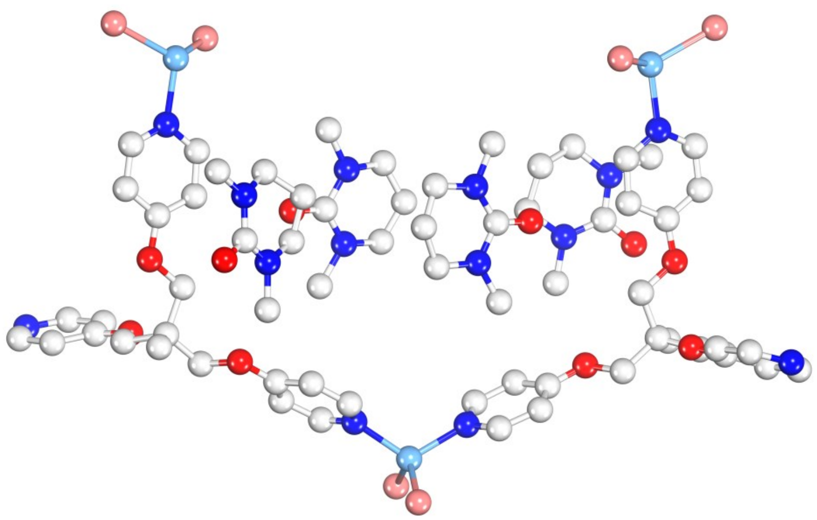

Figure S1 The asymmetric unit of compound **1.**

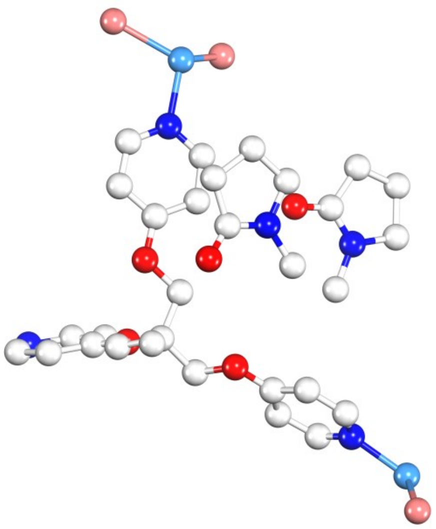

Figure S2 The asymetric unit of compound **2.**

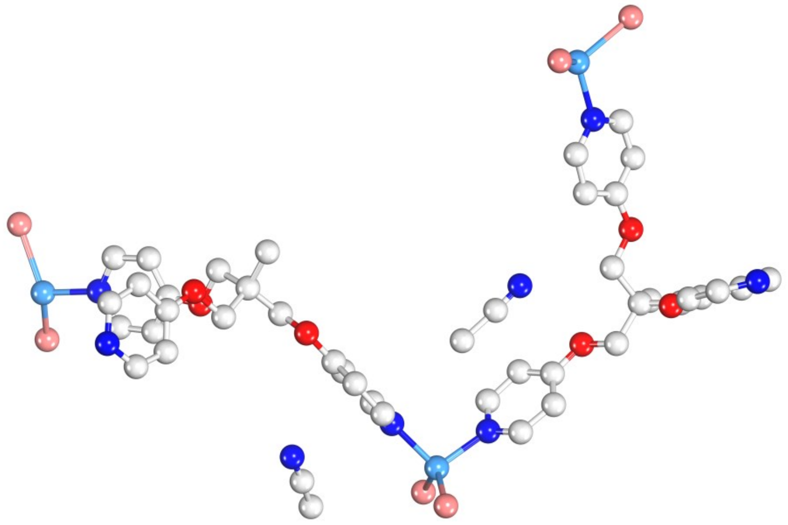

Figure S3 The asymetric unit of compound **3.**

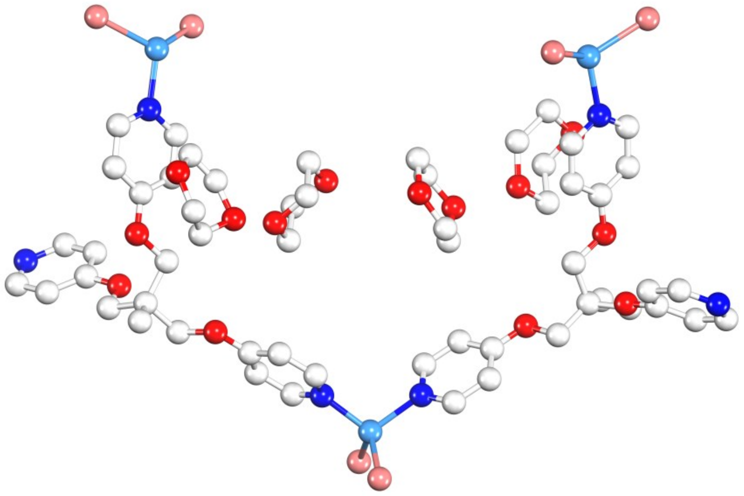

Figure S4 The asymetric unit of compound **4.**

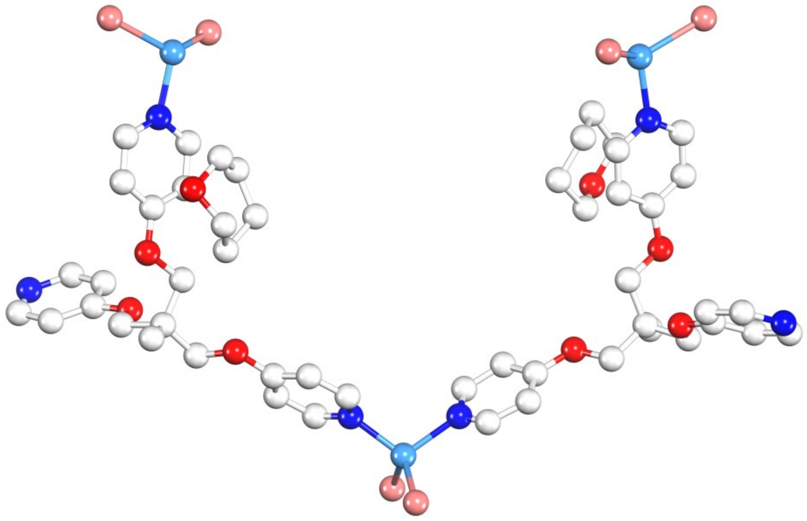

Figure S5 The asymetric unit of compound **5.**

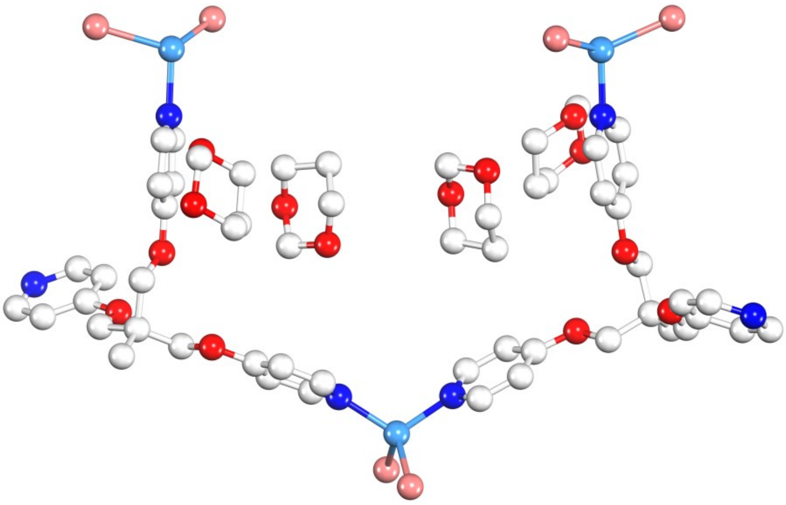

Figure S6 The asymetric unit of compound **6.**

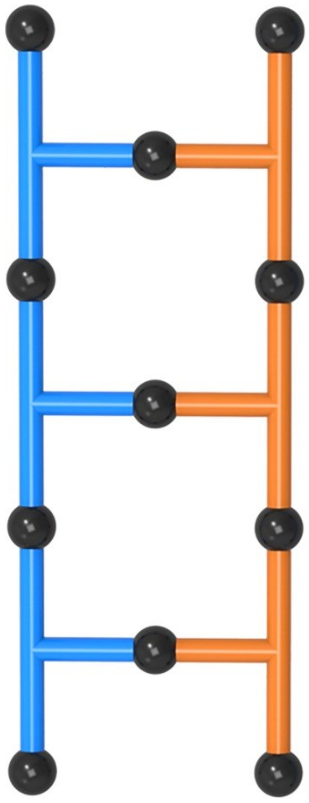

Figure S7 The (4, 3) net of compound **1.**

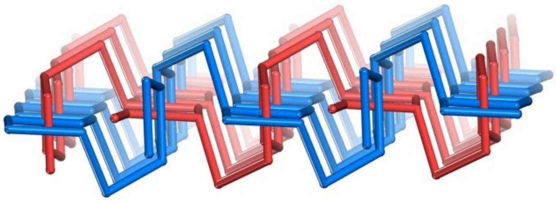

Figure S8 The 2-fold interpenetration comprised in compound **2.**

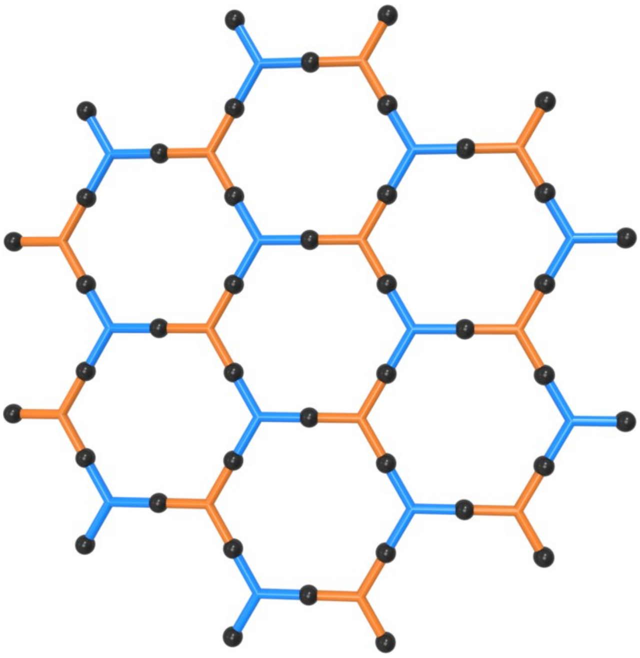

Figure S9 The (6, 3) net from compound **2.**

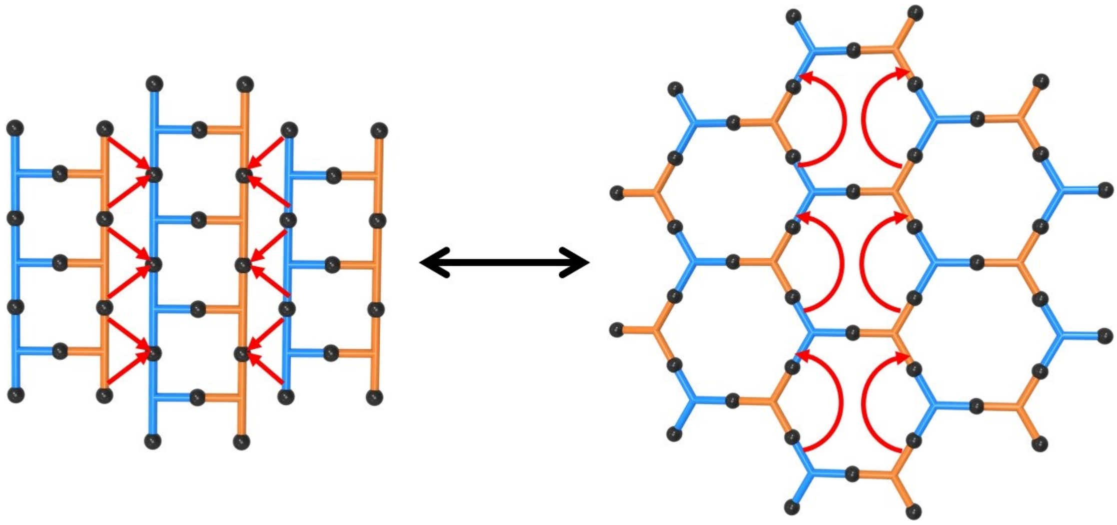

Figure S10 The transformation between(6, 3) net and (4, 3) chain**.**

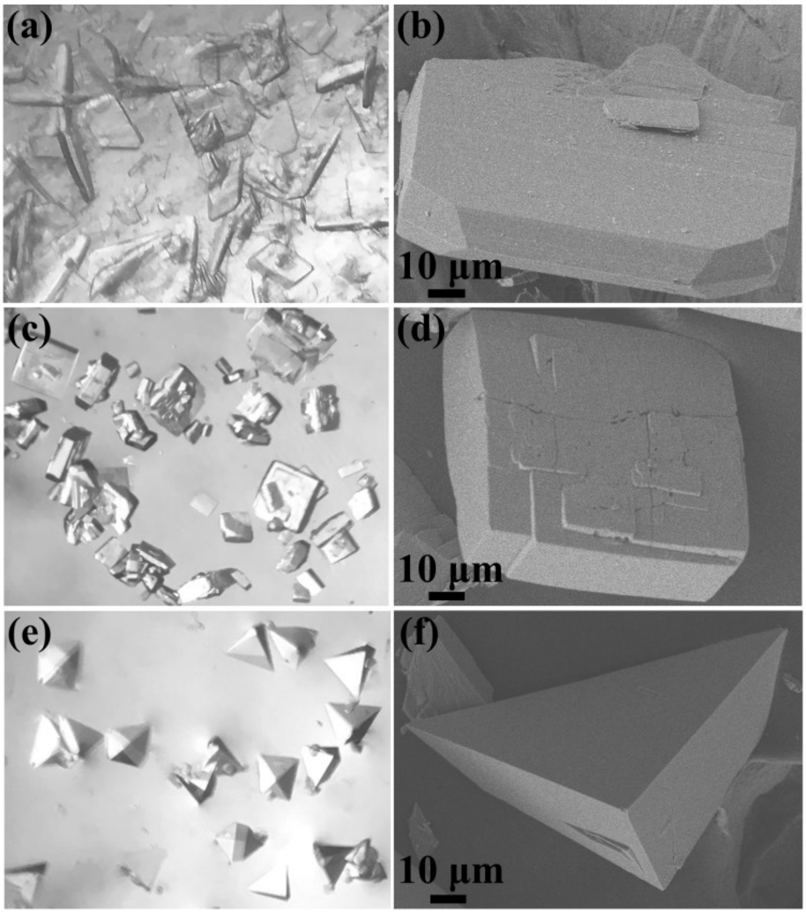

Figure S11 Optical microscopic photos of compounds **1** (a), **2** (c), **3** (e) and the SEM images of compounds **1** (b), **2** (d), **3** (f).

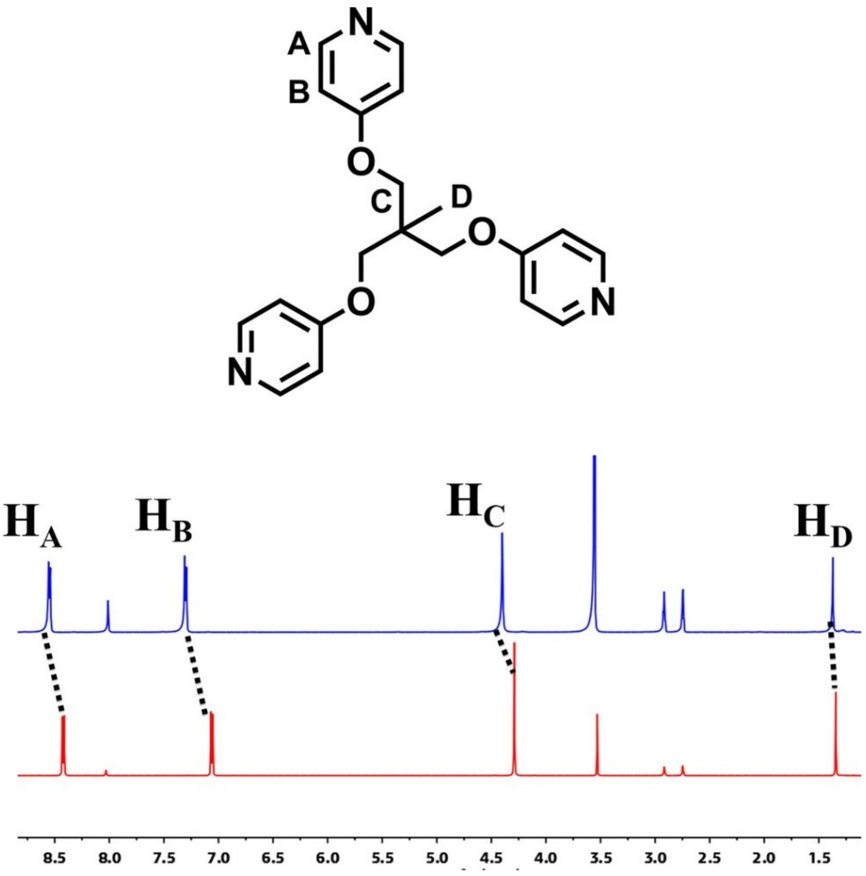

Figure S12 ^{1}H NMR spectrum: red represents the ^{1}H NMR of TPOME ligand; blue represents the ^{1}H NMR of dynamic library.

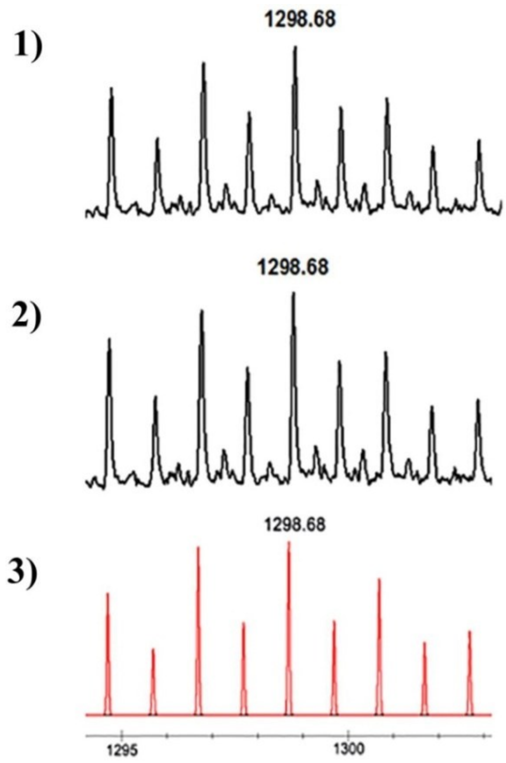

Figure S13 HR-MS spectra: 1) results from sheet-to-tube conversion; 2) results from tube-to-sheet conversion; 3) represents the calculated result.

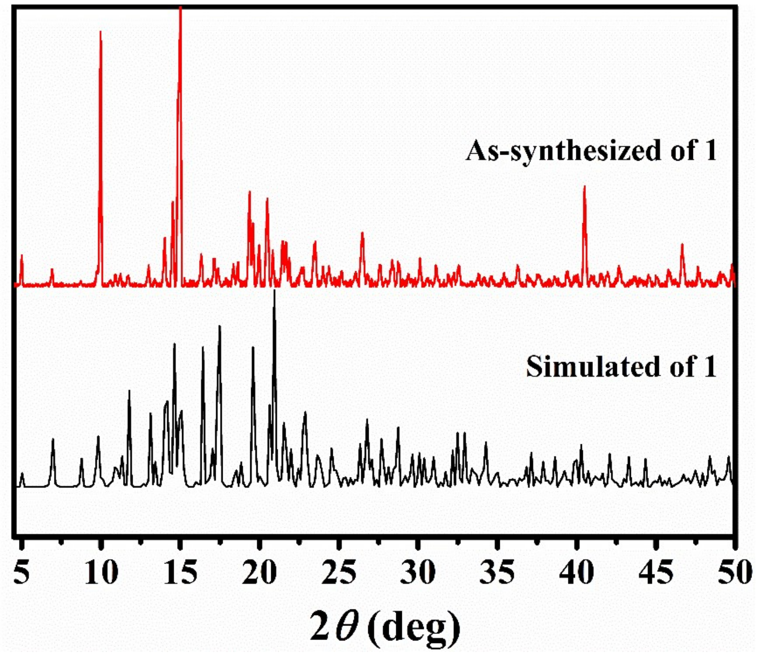

Figure S14 Powder X-ray diffraction of tube **1**. Black: simulated tube **1**; red: as-prepared tube **1**.

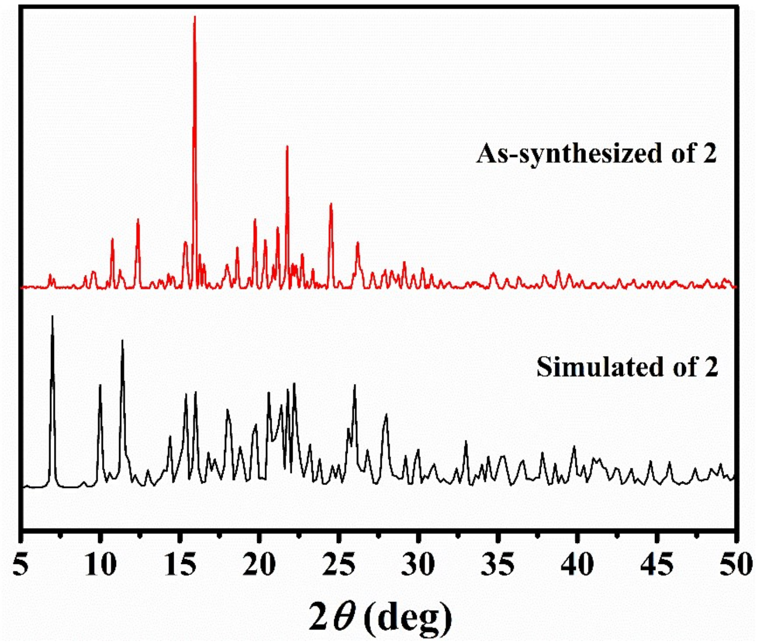

Figure S15 Powder X-ray diffraction of tube **2**. Black: simulated tube **2**; red: as-prepared tube **2**.

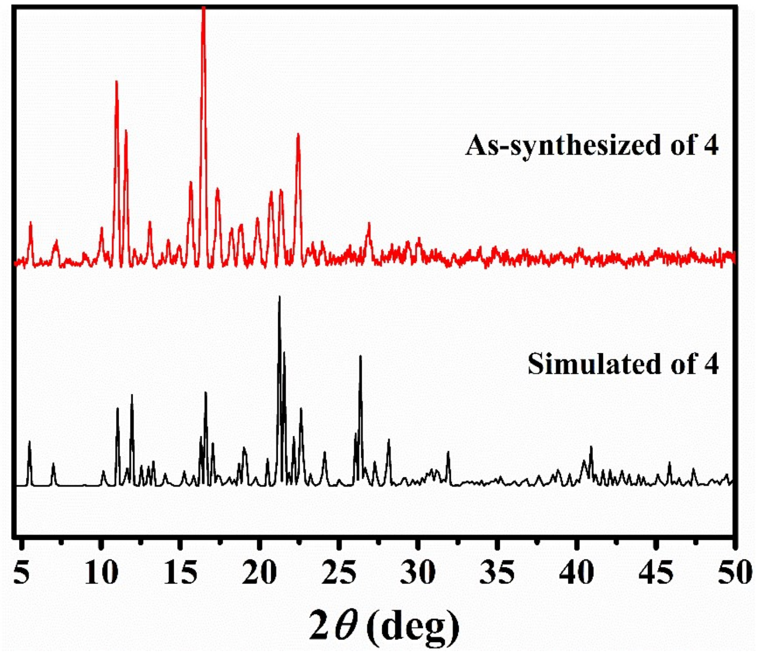

Figure S16 Powder X-ray diffraction of tube **4**. Black: simulated tube **4**; red: as-prepared tube **4**.

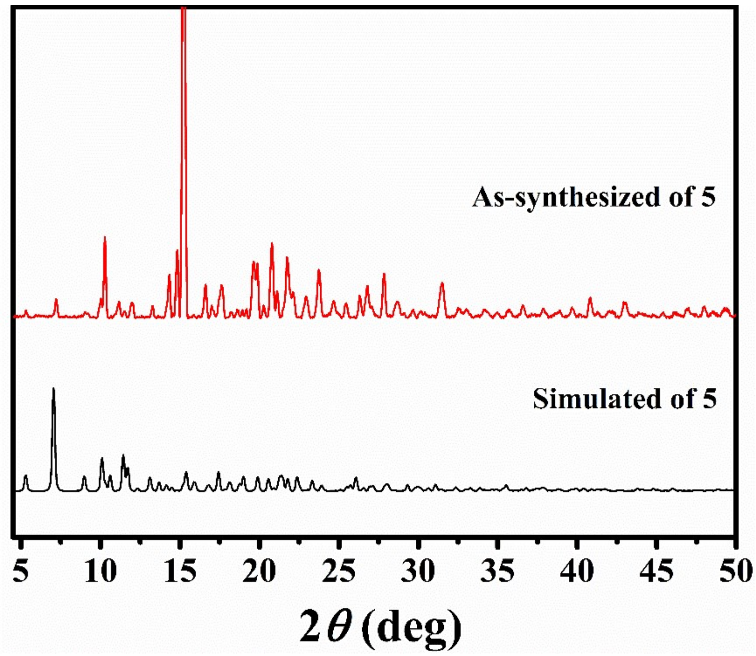

Figure S17 Powder X-ray diffraction of tube **5**. Black: simulated tube **5**; red: as-prepared tube **5**.

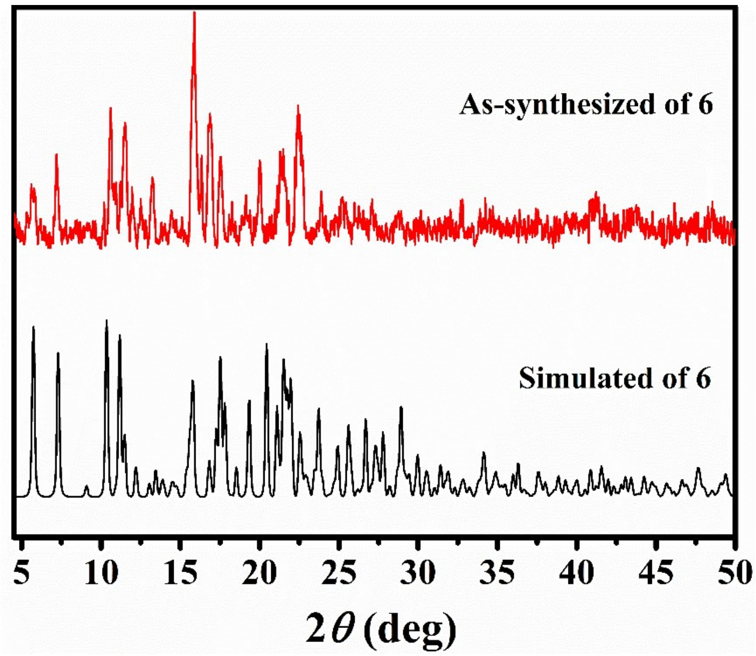

Figure S18 Powder X-ray diffraction of tube **6**. Black: simulated tube **6**; red: as-prepared tube **6**.

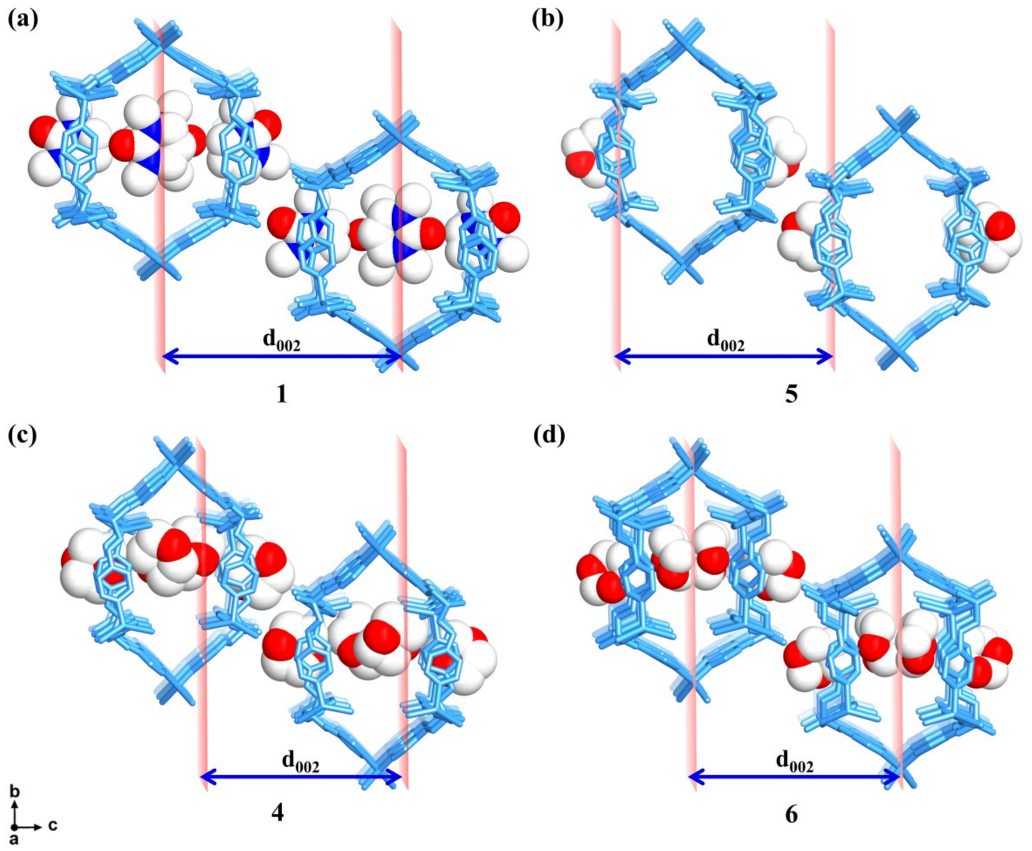

Figure S19 The [002] reflections in the frameworks of tubes **1**, **5**, **4**, **6**. The distances (d_{002}) of neighbour [002] reflections derived from the single crystals X-ray diffraction data of samples **1**, **5**, **4**, **6** is 17.536, 16.645, 16.005, 15.3895, respectively. The d_{002} computed from [002] diffraction peak (2θ) of the powder X-ray diffraction data is about 17.747, 16.868, 15.675, 15.271 for samples **1**, **5**, **4**, **6** separately.

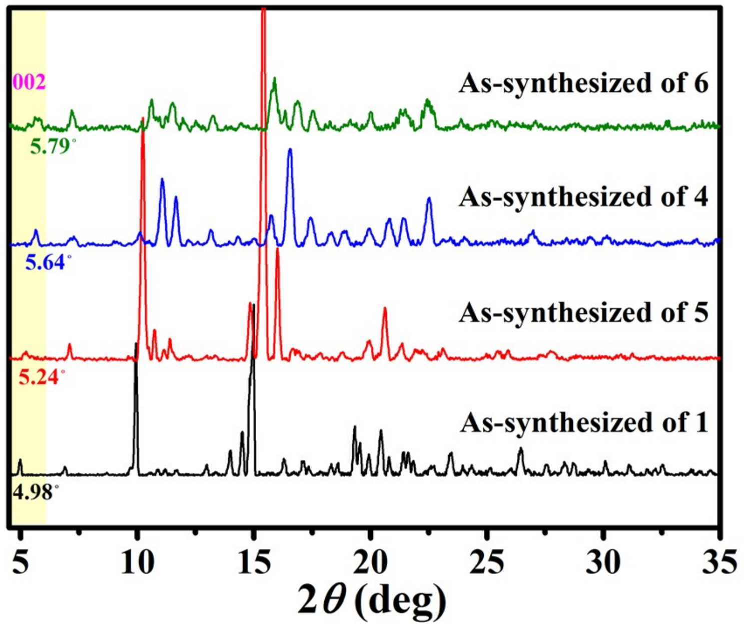

Figure S20 Powder X-ray diffraction of the as-synthesized tubes **1**, **5**, **4**, **6**. The peaks in the yellow rectangular represent the [002] reflections. Black: as-prepared tube **1**; red: as-prepared tube **5**; blue: as-prepared tube **4**; olive: as-prepared tube **6.**

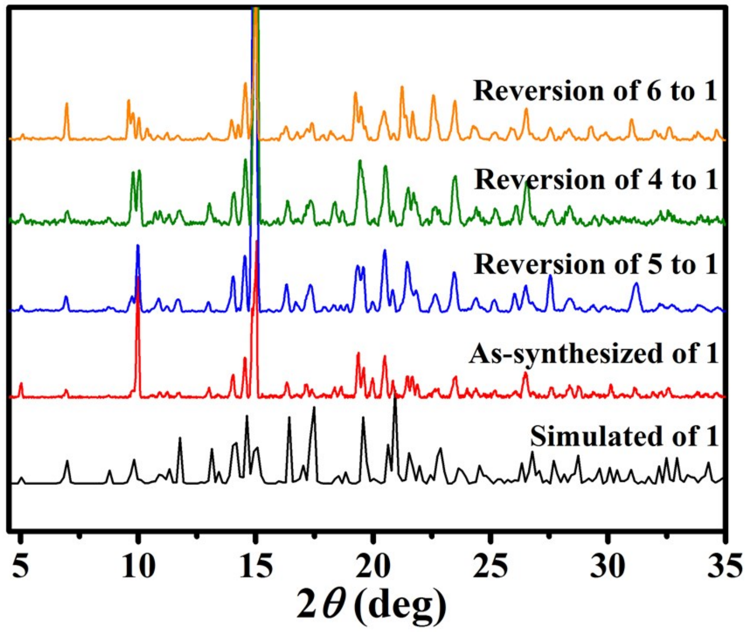

Figure S21 Powder X-ray diffraction of the recovered samples after re-soaking tubes **5**, **4**, **6** into DMPU solution. Black: simulated tube **1**; red: as-prepared tube **1**; blue: re-soaking the tetrahydropyrane-encapsulated tube **5** in DMPU solution; olive: re-soaking the 1, 4-dioxane-encapsulated tube **4** in DMPU solution; orange: re-soaking

the 1, 3-dioxane-encapsulated tube **6** in DMPU solution.

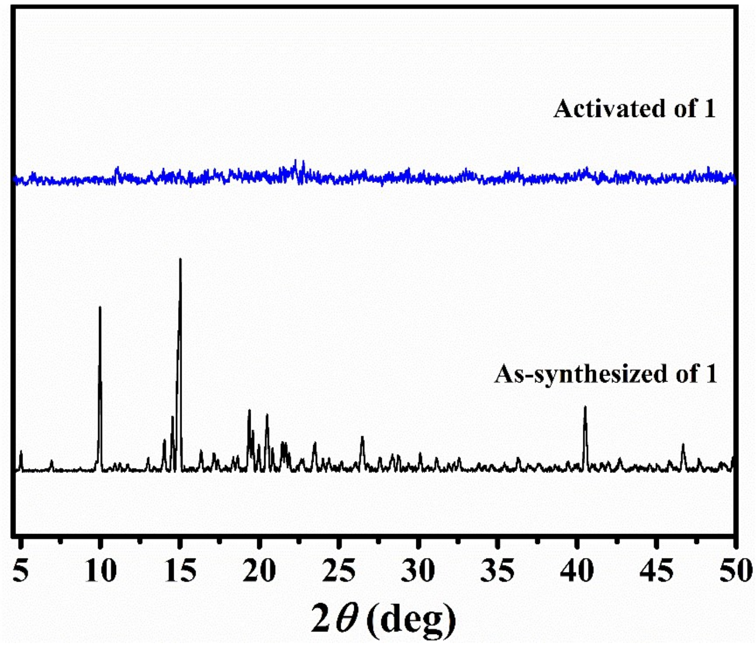

Figure S22 Powder X-ray diffraction of tube **1** after activated. Black: as-prepared tube 1; blue: after activated.

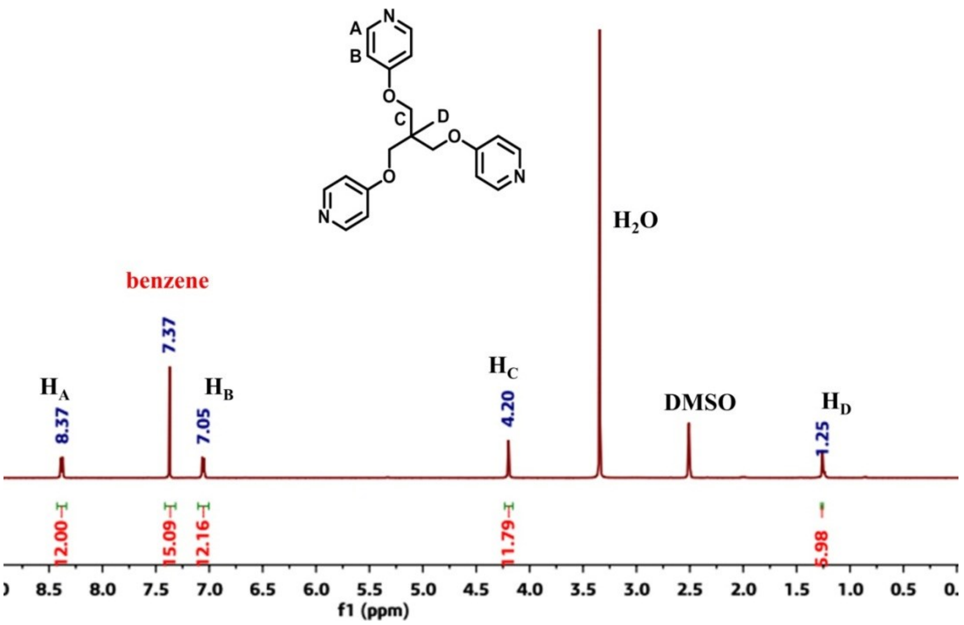

Figure S23 ^{1}HNMR (DMSO-d6) of benzene-encapsulated sample (compound **7**) .

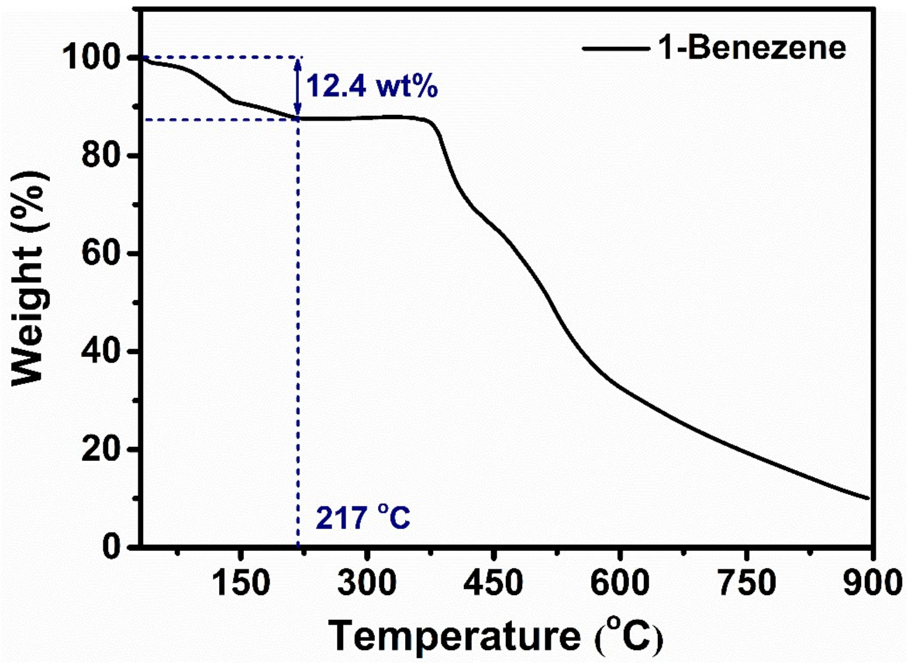

Figure S24 Thermogravimetric analysis curves of benzene-encapsulated sample (compound **7**).

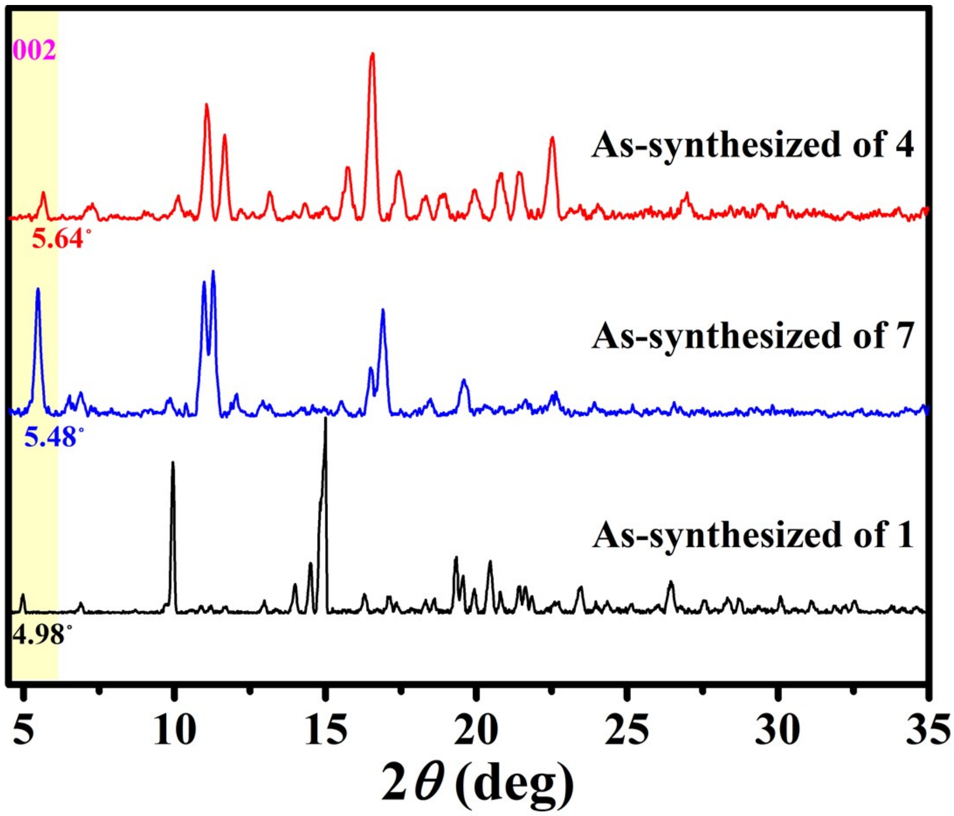

Figure S25 Powder X-ray diffraction of the benzene-encapsulated sample (Compound **7**). Black: as-prepared tube **1**; blue: after soaking tube **1** in benznene solution; red: as-prepared tube **4**.

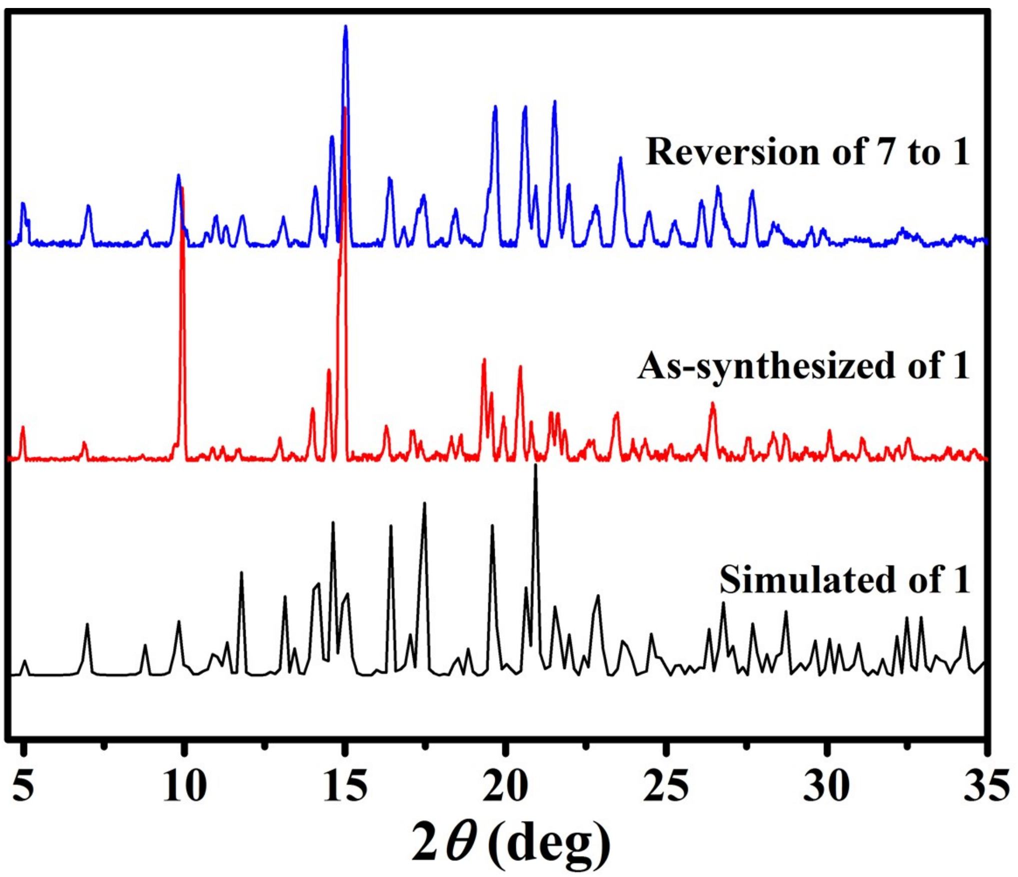

Figure S26 Powder X-ray diffraction of the recovered samples after re-soaking Compound 7 into DMPU solution. Black: simulated tube 1; red: as-prepared tube 1; blue: re-soaking the benzene-encapsulated Compound 7 in DMPU solution.

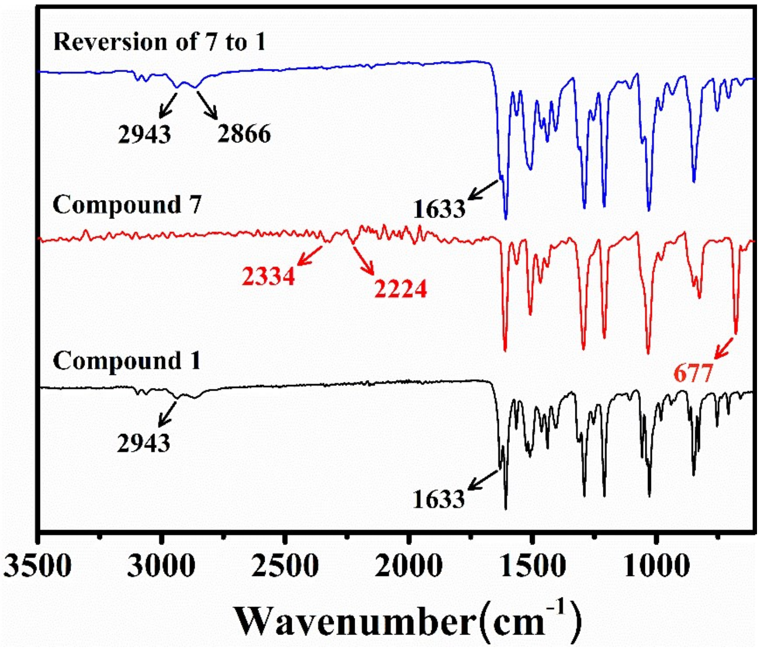

Figure S27 FT-IR spectra of compound **1**, compound **7** and the recovered samples after re-soaking compound **7** into DMPU solution. Black: compound **1**; red: compound **7**; blue: re-soaking the benzene-encapsulated compound **7** in DMPU solution. The peak at 2943, 2866 and 1666 cm^{-1} were the characteristic peaks of DMPU in compound **1**. The peaks at 2334, 2224 and 677 cm^{-1} were the characteristic peak of benzene in compound **7**.

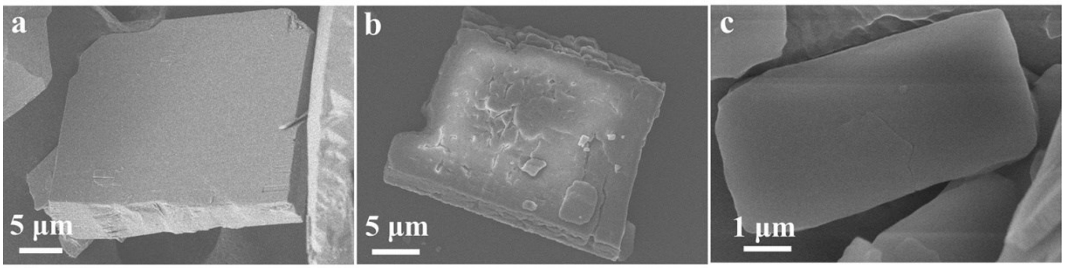

Figure S28 SEM images of (a) compound **1**, (b) compound **7** and (c) restored compound **1** by re-soaking the benzene-encapsulated compound **7** in DMPU solution.

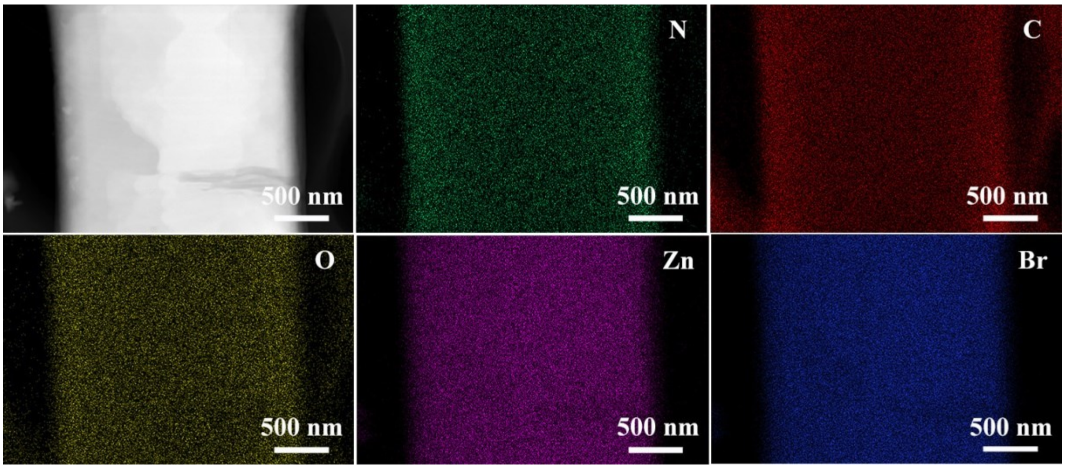

Figure S29 The HAADF-STEM images and corresponding elemental mapping of compound **1**.

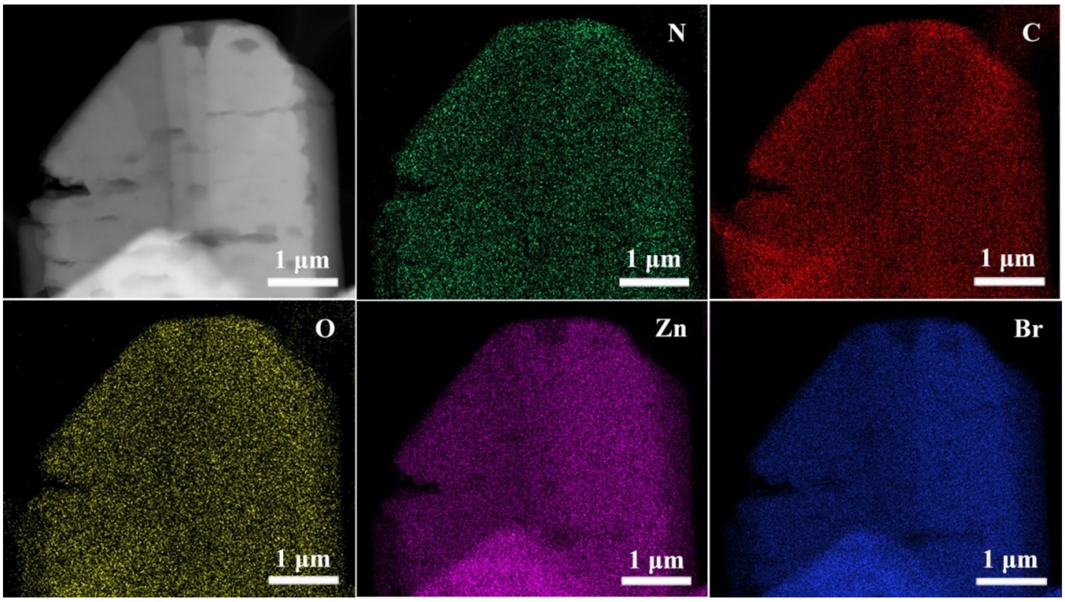

Figure S30 The HAADF-STEM images and corresponding elemental mapping of compound **7**.

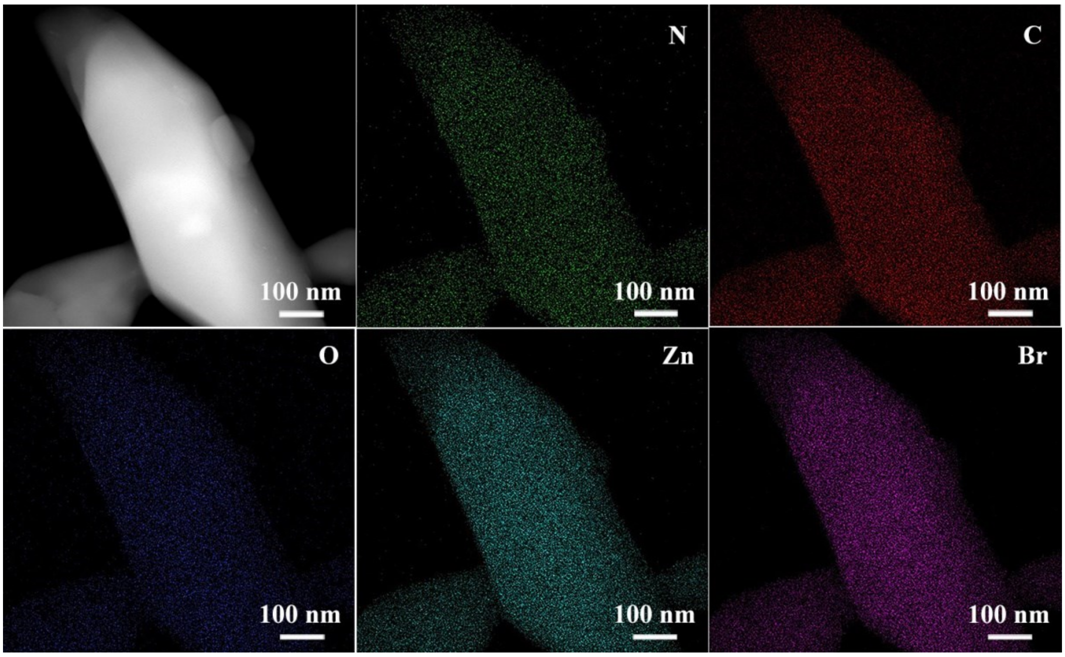

Figure S31 The HAADF-STEM images and corresponding elemental mapping of the restored compound **1** by re-soaking the benzene-encapsulated compound **7** in DMPU solution.

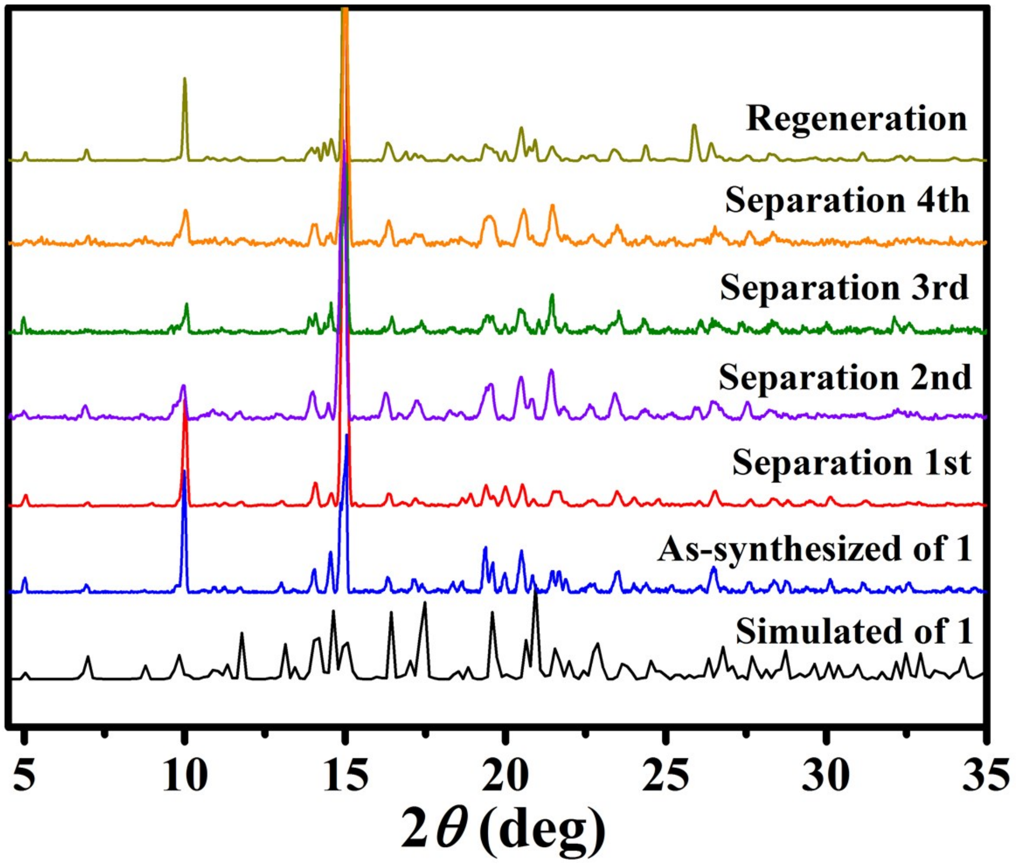

Figure S32 Powder X-ray diffraction of tube **1** in the separation process. Black: simulated tube **1**; blue: as-prepared tube **1**; red: after the first separation cycle; violet: after the second separation cycle; olive: after the third separation cycle; orange: after the forth separation cycle; dark yellow: the regeneration of tube **1**. (for comparison, the used tube **1** are resoaked in a small amount of DMPU solution after separation process).

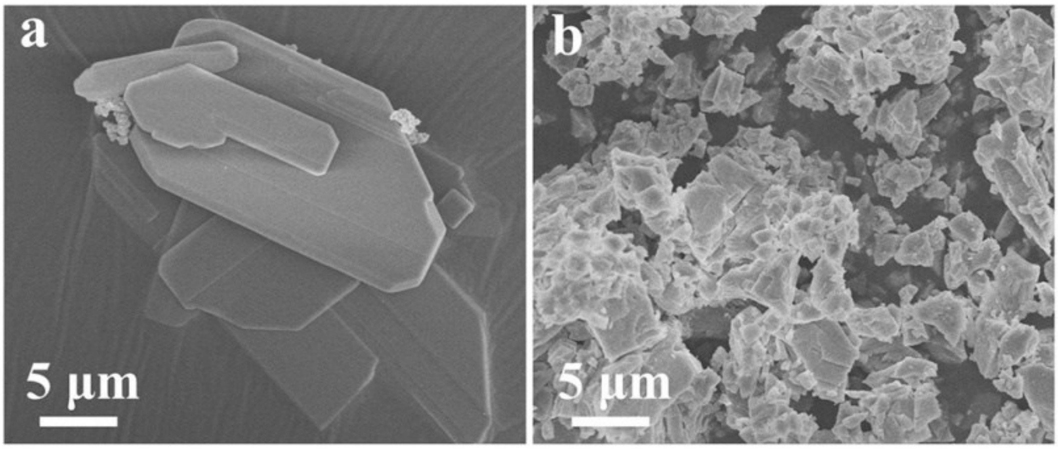

Figure S33 SEM images of (a) tube **1**, (b) tube **1** after four separation cycles.

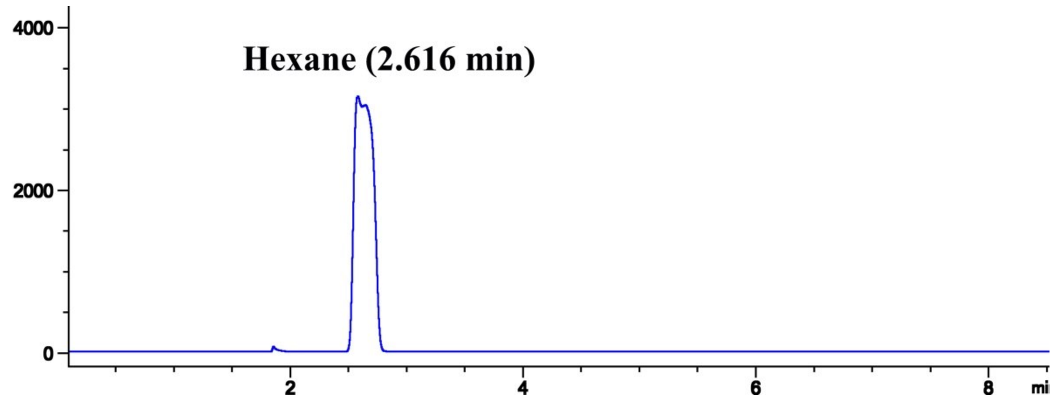

Figure S34 The retention time of hexane.

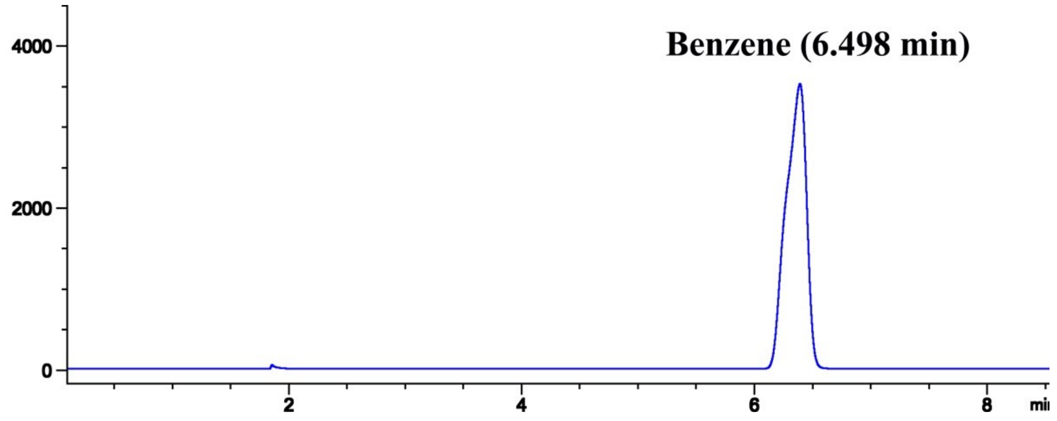

Figure S35 The retention time of benzene.

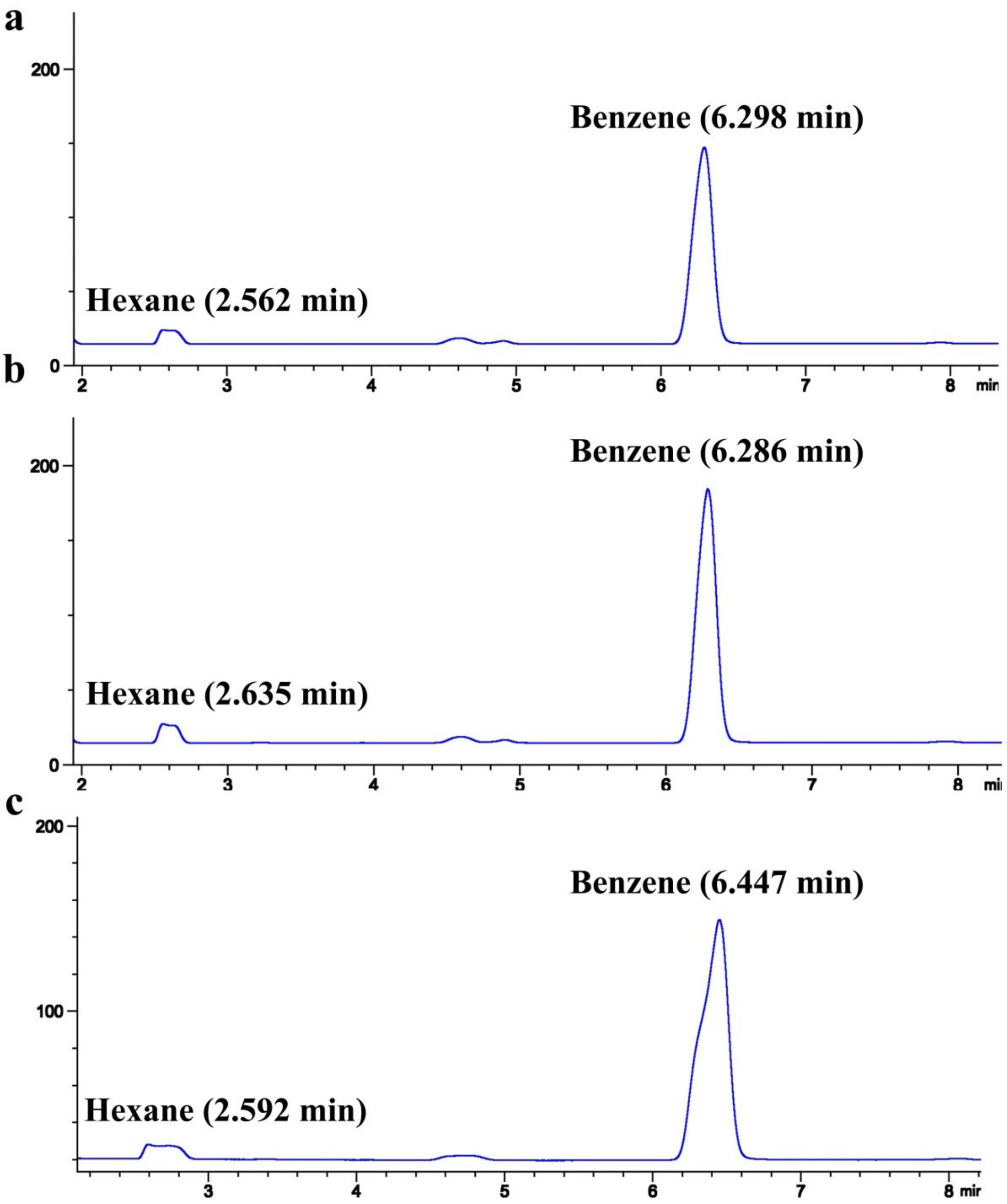

Figure S36 The Gas chromatogram of the first separation.

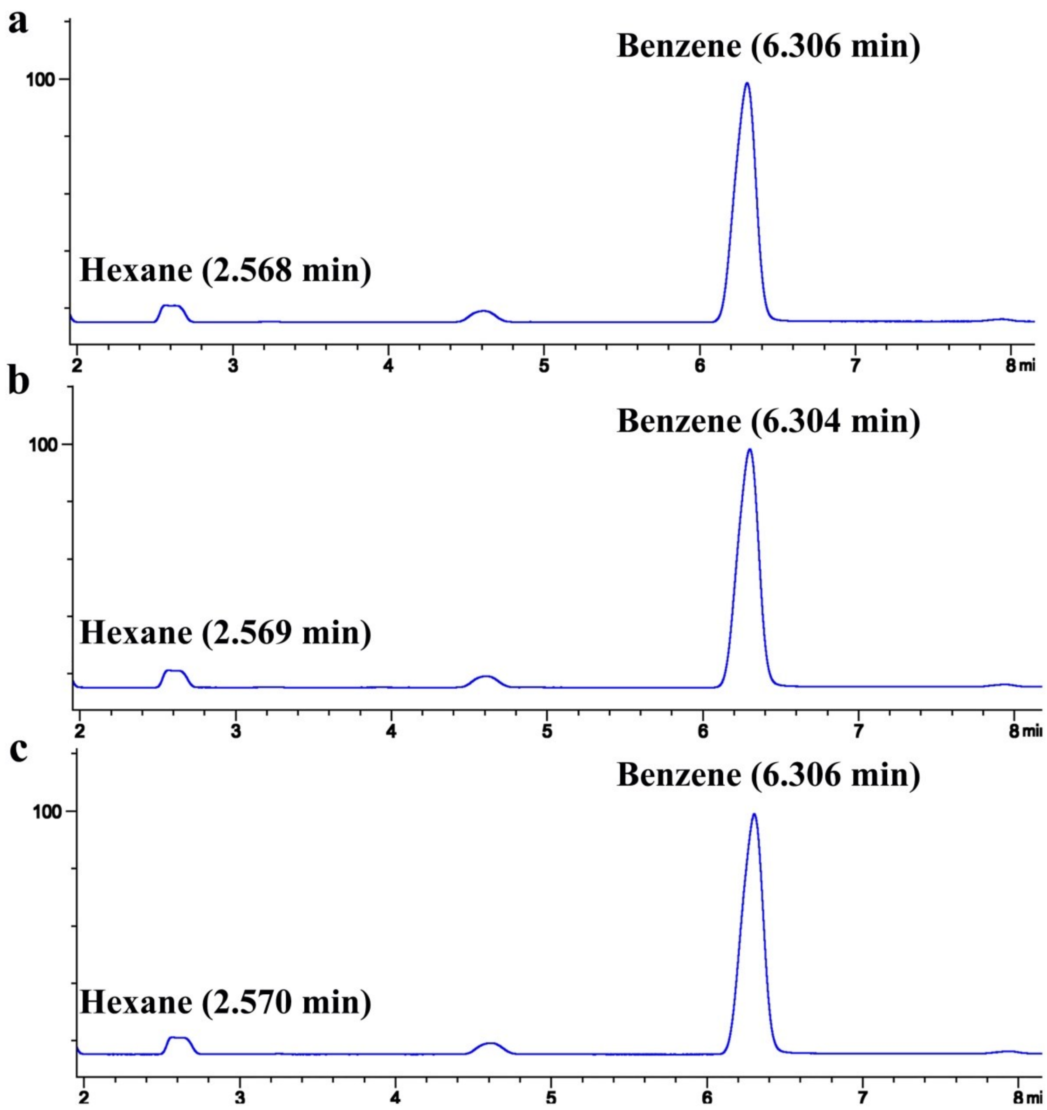

Figure S37 The Gas chromatogram of the second separation.

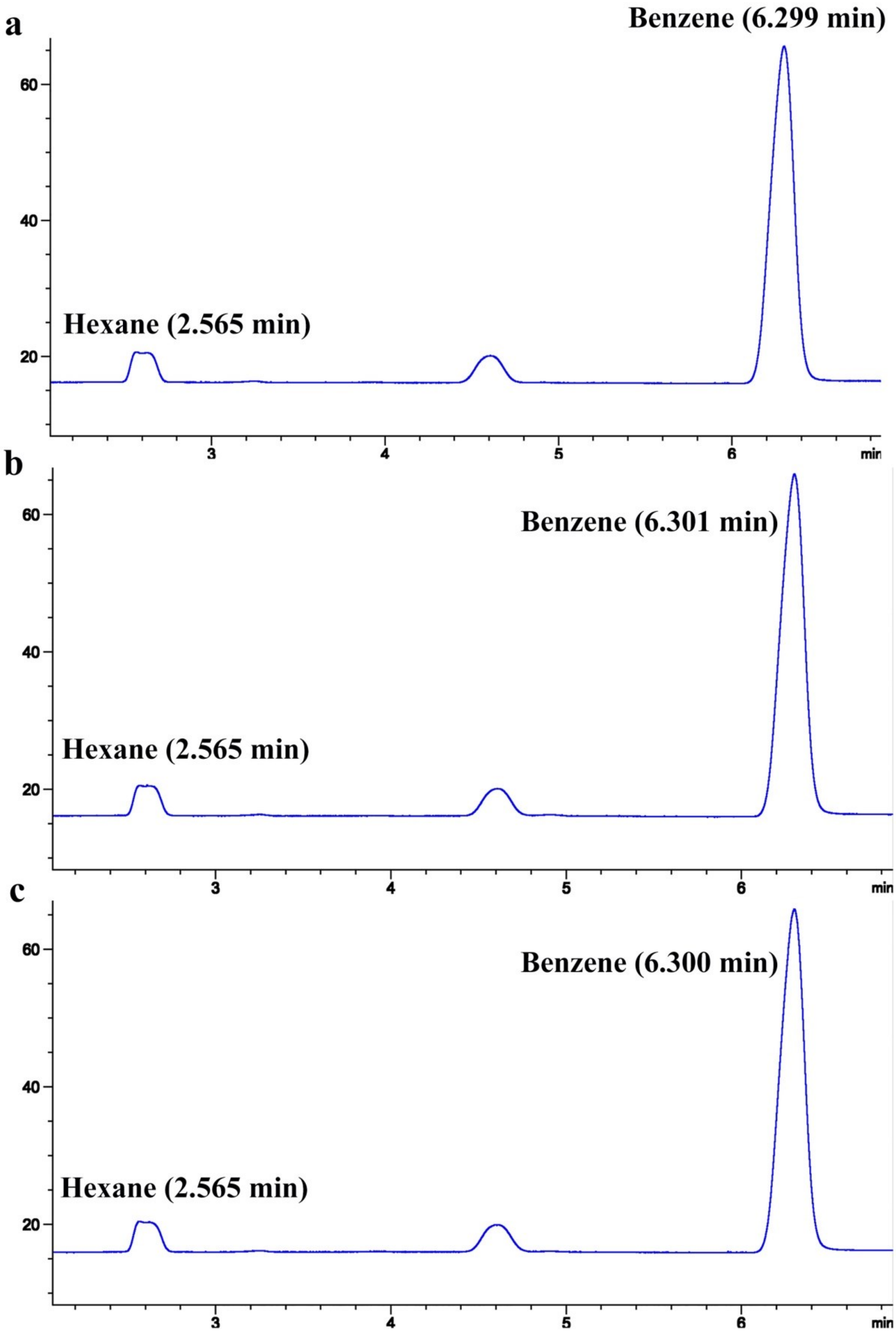

Figure S38 The Gas chromatogram of the third separation.

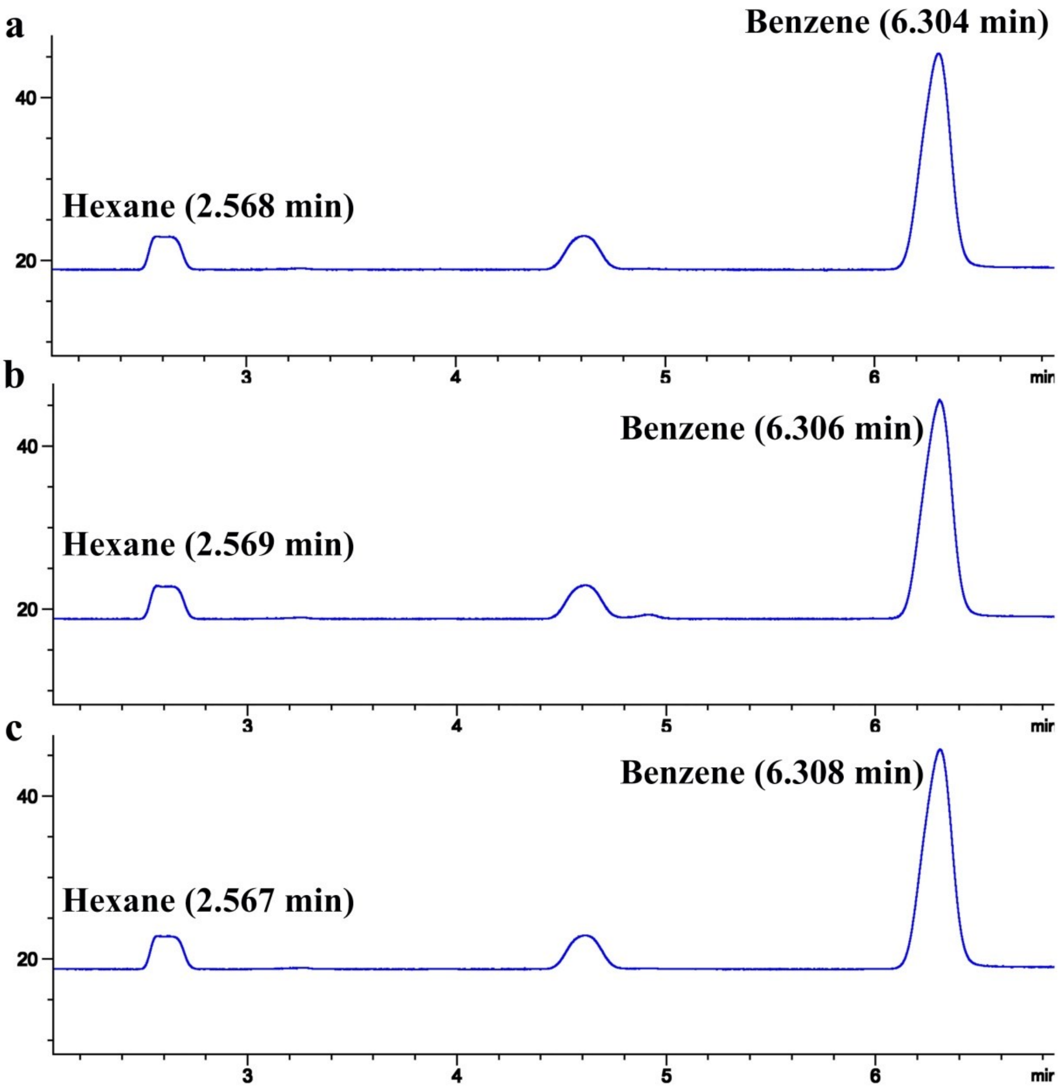

Figure S39 The Gas chromatogram of the forth separation.

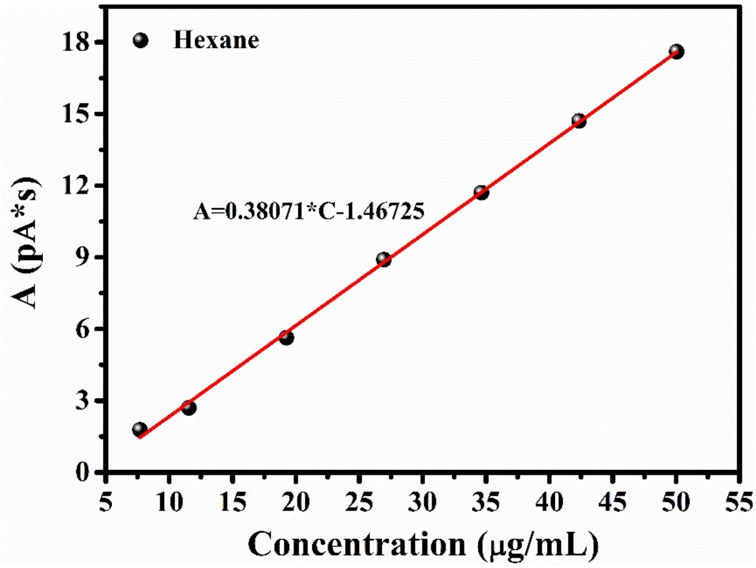

Figure S40 The calibration curve of hexane.

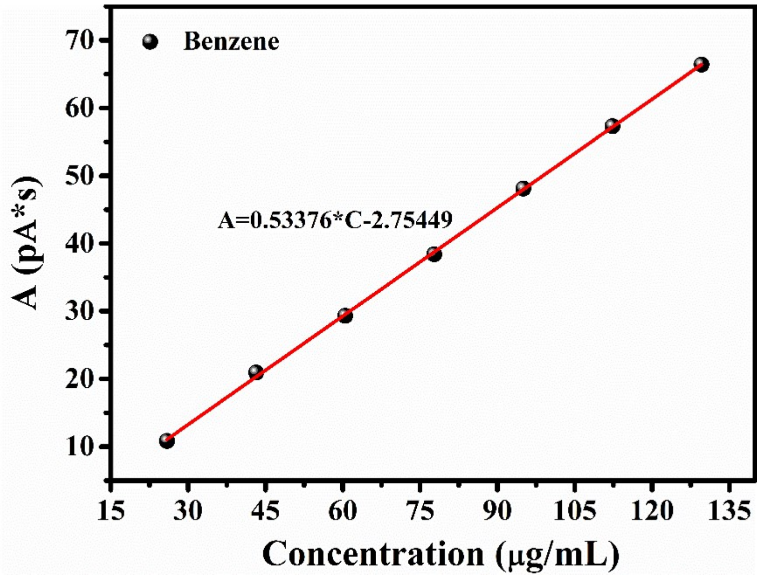

Figure S41 The calibration curve of benzene.

Table S5. The 1st-4th separation ratios (calculated from Gas chromatogram Figure S36-39 and calibration curves Figure S40-41).

Separation	Area of hexane	Area of benzene	Separation ratio	Average	Standard Deviation
1st	4.63	73.52	90.57%	89.62%	0.95
	5.76	78.58	89.63%		

	6.25	75.83	88.66%		
	2.92	43.13	88.93%		
2nd	3.25	39.14	87.21%	88.10%	0.86
	2.88	39.4	88.16%		
	2.08	23.00	84.77%		
3rd	2.51	23.26	83.41%	84.69%	1.24
	2.10	23.55	85.89%		
	1.98	12.79	77.57%		
4th	2.24	12.67	76.17%	77.42%	1.19
	2.00	13.76	78.53%		

References:

1. G. M. Sheldrick, *SHELXTL-97,* Program for the Solution of Crystal Structures, University of Göttingen, Germany, **1997**.
2. A. L. Spek, Single-crystal structure validation with the program *PLATON. J. Appl. Crystallogr.*, **2003**, *36*, 7-13.
3. P. Van Der Sluis, A. L. Spek, BYPASS: an effective method for the refinement of crystal structures containing disordered solvent regions. *Acta Crystallogr., Sect. A*, **1990**, *46*, 194-201.
